# Effects of flame made zinc oxide particles in human lung cells - a comparison of aerosol and suspension exposures

**DOI:** 10.1186/1743-8977-9-33

**Published:** 2012-08-17

**Authors:** David O Raemy, Robert N Grass, Wendelin J Stark, Christoph M Schumacher, Martin JD Clift, Peter Gehr, Barbara Rothen-Rutishauser

**Affiliations:** 1 Adolphe Merkle Institute, Bionanomaterials, University of Fribourg, Rte de l’Ancienne Papeterie, P.O. Box 209, CH-1732, Marly, Switzerland; 2 Functional Materials Laboratory, Institute for Chemical and Bioengineering, Wolfgang-Pauli-Str. 10, ETH Zurich, HCI E 105, CH-8093, Zurich, Switzerland; 3 Institute of Anatomy, Division of Histology, University of Bern, Baltzerstrasse 2, CH-3000 Bern 9, Bern, Switzerland; 4 Respiratory Medicine, Bern University Hospital, Inselspital, Freiburgstrasse, CH-3010, Bern, Switzerland

**Keywords:** Zinc oxide particles, Aerosol exposure, Air liquid interface (ALI), Suspension exposure

## Abstract

**Background:**

Predominantly, studies of nanoparticle (NPs) toxicology *in vitro* are based upon the exposure of submerged cell cultures to particle suspensions. Such an approach however, does not reflect particle inhalation. As a more realistic simulation of such a scenario, efforts were made towards direct delivery of aerosols to air-liquid-interface cultivated cell cultures by the use of aerosol exposure systems.

This study aims to provide a direct comparison of the effects of zinc oxide (ZnO) NPs when delivered as either an aerosol, or in suspension to a triple cell co-culture model of the epithelial airway barrier. To ensure dose–equivalence, ZnO-deposition was determined in each exposure scenario by atomic absorption spectroscopy. Biological endpoints being investigated after 4 or 24h incubation include cytotoxicity, total reduced glutathione, induction of antioxidative genes such as heme-oxygenase 1 (HO–1) as well as the release of the (pro)-inflammatory cytokine TNFα.

**Results:**

Off-gases released as by-product of flame ZnO synthesis caused a significant decrease of total reduced GSH and induced further the release of the cytokine TNFα, demonstrating the influence of the gas phase on aerosol toxicology. No direct effects could be attributed to ZnO particles. By performing suspension exposure to avoid the factor “flame-gases”, particle specific effects become apparent. Other parameters such as LDH and HO–1 were not influenced by gaseous compounds: Following aerosol exposure, LDH levels appeared elevated at both timepoints and the HO–1 transcript correlated positively with deposited ZnO-dose. Under submerged conditions, the HO–1 induction scheme deviated for 4 and 24h and increased extracellular LDH was found following 24h exposure.

**Conclusion:**

In the current study, aerosol and suspension-exposure has been compared by exposing cell cultures to equivalent amounts of ZnO. Both exposure strategies differ fundamentally in their dose–response pattern. Additional differences can be found for the factor time: In the aerosol scenario, parameters tend to their maximum already after 4h of exposure, whereas under submerged conditions, effects appear most pronounced mainly after 24h. Aerosol exposure provides information about the synergistic interplay of gaseous and particulate phase of an aerosol in the context of inhalation toxicology. Exposure to suspensions represents a valuable complementary method and allows investigations on particle-associated toxicity by excluding all gas–derived effects.

## Background

The extensive utilization of gas phase processes and special flame synthesis techniques to produce particles in both, the industrial and academic environment, raises concerns as to the exposure risk of workers, laboratory personnel and scientists to airborne particles, and the potential adverse health effects arising therefrom [1-3]. The market for flame made particles has been shaped most notably by carbon black and ceramic powders such as pigmentary titanium dioxide (TiO_2_) and fumed silica (SiO_2_), but also other single oxide materials such as Cerium -, Iron – and Zinc oxide (ZnO) are produced in commercial quantities [4]. Inhalation is considered as the most likely route of exposure to such airborne particulate matter in occupational settings [1,3,5]. As a consequence, aerosol deposition on the respiratory system has gained considerable attention in recent years and has been subject of extensive modelling [3,6,7]. A health risk, however, may only arise if both, a hazard potential of the particles as well as exposure exists. Therefore studies of workplace related exposure of airborne, engineered (nano) particles were initiated (reviewed i.a. in [8]). With special focus on gas – phase processes, exposure levels have been assessed during production of metal and composite particles by flame spray synthesis in an university laboratory [9], during the coating of glass compounds with CeO_2_ particles derived from a flame spray process [10] and in a pilot scale particle production site [11]. As more general approach, Methner *et al.* evaluated aerosol emissions caused by handling or synthesis of a set of different nanomaterials (e.g. fullerene, carbon nanotubes, quantum dots and metal oxides) in several research and development laboratories and manufacturing sites [5]. As demonstrated by the literature, occupational exposure to airborne particulate matter (primary particles, aggregates and/or agglomerates) can be considered as inevitable within industrial and academic settings. As a scientific base in order to understand, predict and manage potential health risks, the intrinsic hazard potential of these materials must be addressed in the context of the lung [12]. In inhalation toxicology, two fundamentally different *in vitro* testing strategies have emerged, both present system immanent advantages and disadvantages (reviewed in [12]). Lung cells can be exposed to particles either i.) under submerged conditions by covering the cultures with a particle suspension in growth medium ii.) or by applying an aerosol to air – liquid interface (ALI) cultivated cell cultures. The suspension scenario originates from classical toxicity testing of chemicals and is widely used within nanotoxicology due to its ease of handling, high – throughput rate, reproducibility, the possibility to observe cells over comparable long timespans, and the ability to compare obtained results with a plethora of already available studies [12]. However, several system immanent limitations have to be considered, such as the requirement of collecting and resuspending the (nano) particle sample. Furthermore, the cell culture medium can interact with the dispersed particles (colloids) in a hardly predictable manner, resulting in system immanent effects such as specific and unspecific surface coating with medium components (protein corona), change in surface activity and particle dissolution processes [13] (e.g. effect of ionic species) [14]. In addition, medium properties (*i.e.* protein content, ionic strength, viscosity, density, pH) interfere with diffusion and sedimentation driven particle motion (particokinetics) and shape agglomeration processes [15-17]. To avoid such artificial “matrix effects” originating from culture medium, novel direct aerosol exposure systems have been designed which consider aerosols as multifactorial systems, composed of gas and therein dispersed particle components [12,18-22]. Hence, *in vitro* exposure of aerosols at the air liquid interface (ALI) represents a more realistic exposure scenario as it preserves the physical and chemical characteristics of airborne particles [23].

The understanding of the influence of the particle surrounding, either gas phase or liquid medium, on particle behavior and toxicity is a strategic question and demands investigation. Although frequently described as being a more physiologic method, there have been only a limited number of studies on the direct comparison of ALI exposure with “conventional” suspension exposure [21,24,25].

The aim of this study therefore, was to evaluate the *in vitro* toxicology of a representative metal oxide (nano) particle aerosol under workplace conditions, and to compare its adverse effects with results from a dose - equivalent suspension exposure scenario.

With regard to its comparably well - established toxicological background and its potential airborne release into working environments (flame synthesis), ZnO has been chosen for this comparative study. It is widely used in classical industrial chemistry [26,27] and nanostructured ZnO particles have attracted much attention in the recent years due to their unique optical and electrical properties [28] making them e.g. key ingredients for modern sunscreens [29]. ZnO particles are toxic within several different cell types; *In vitro*, they have been shown to cause oxidative stress [30-33], to trigger an anti-oxidative response [30,34], to promote inflammation [30,35-37] and to elicit cytotoxic effects [13,30-32,38]. Furthermore, occupational medicine has long - standing experience with “metal fume fever”, a classical flu like inflammatory disease among workers exposed to metal oxide, mainly ZnO, aerosols (“fumes”) released during such processes as welding, heating and cutting of galvanized metals [29,39-41].

The hazard potential of airborne ZnO was assessed *in vitro* by the use of a recently established experimental approach, which enables the simulation of accidental aerosol release in occupational settings [15,22,42,43]. This exposure system directly combines *in situ* aerosol production by flame spray pyrolysis with simultaneous particle deposition from the gas – phase onto air – liquid interface cultivated lung cell cultures. By placing particle source and exposure site within the same well stirred compartment (glove box), aging processes such as agglomeration, chemical modifications etc. take place in a realistic and predictable manner, particles transport follows well understood principles (passive motion by diffusion and sedimentation) [44] and the influence of flame off – gas compounds can be assessed. An advanced 3D triple cell co – culture model of the human epithelial airway barrier was used for this study. It consists of a confluent layer of 16HBE14o- bronchial epithelial cells, in co-culture with human blood monocyte derived macrophages on the apical side and human blood monocyte derived dendritic cells on the basal side of a culture insert [45,46]. Dependent on the exposure scenario, cells were either cultivated under submerged conditions, or kept at ALI. Based on the current mechanistic understanding of ZnO toxicity this study included biological endpoints such as acute cytotoxicity (LDH assay), the release of (pro)-inflammatory mediators, cellular antioxidant capacity (GSH content) and the transcription of antioxidative genes (superoxide dismutase 1 (SOD1) and heme oxygenase 1 (HO-1)).

The experimental setup was the following. Different ZnO aerosols (doses) were produced by a flame-reactor, operated for 0 (flame off-gas control), 22, 45 and 90 seconds (sec). ALI cultivated cell cultures were exposed to the airborne particles for 30 min, followed by 4 or 24 hours (h) post-incubation. Cell cultures placed in a separate incubator during exposure experiments served as external “incubator control” (baseline). Supernatants were taken and stored from the upper well.

Suspension exposure experiments were carried out in a dose range comparable to the aerosol scenario, with 4 and 24 h incubation time. Supernatant samples were taken from the upper (apical) and the lower (basal) compartment (well) of the cell culture insert. To ensure a comparable dosimetry (overlapping dose range) in both scenarios, the mass deposition per area was determined by atomic absorption spectroscopy (AAS). The parameters of the aerosol scenario (exposure time and reactor runtime) were chosen to reflect a realistic hazard situation in an occupational setting as close as possible.

## Results

### Aerosol scenario

#### Aerosol characterization

**Figure 1:**
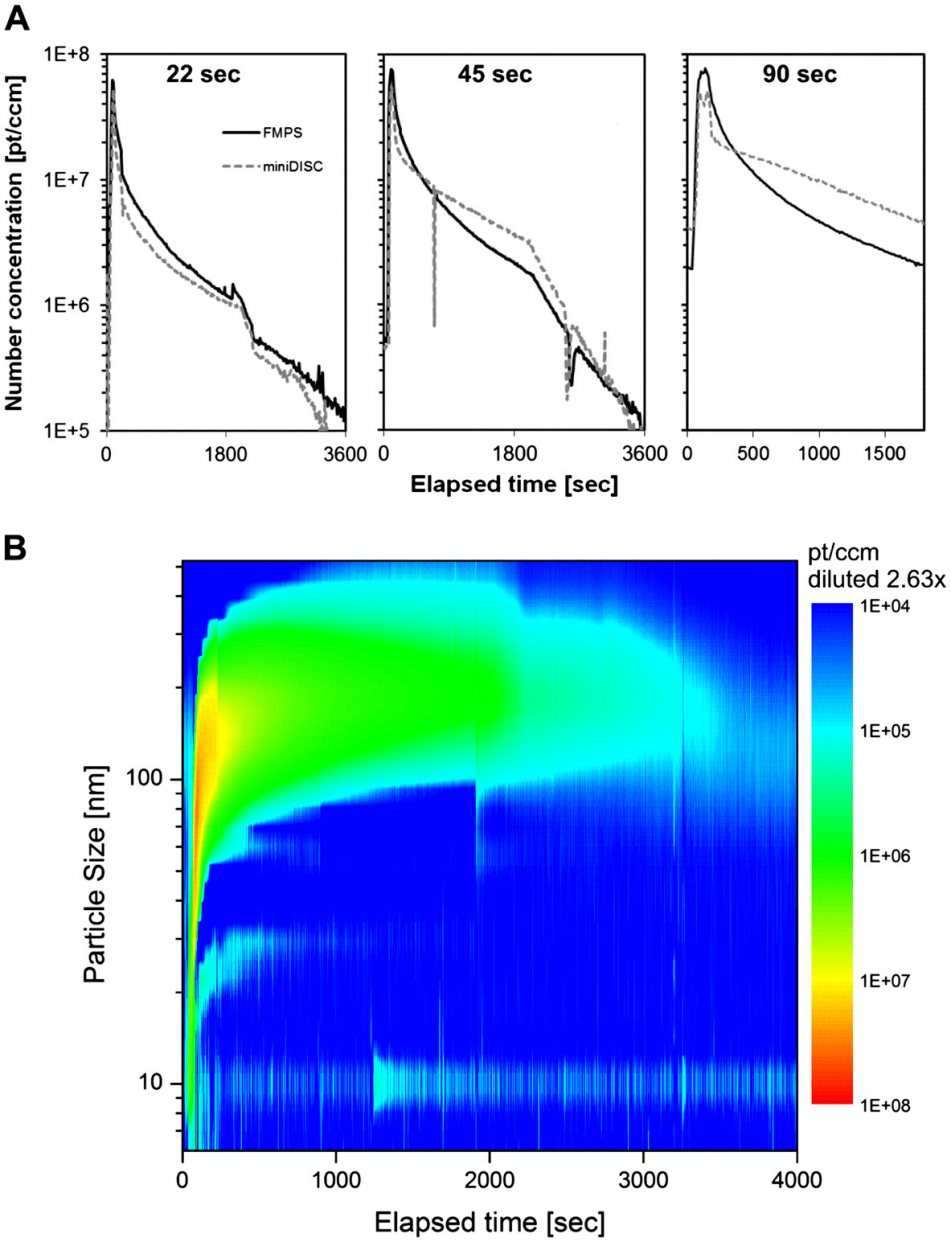
**Aerosol characterization. A**. The total aerosol number concentration was determined in parallel by two measuring devices (FMPS and a miniDiSC) for experimental runs with 22, 45 and 90 sec ZnO production (dataset averaged over a 10 sec interval). The cell cultures were exposed to the airborne particles for 30 min (1800 sec). Shortly after this timespan, the glove box chamber was flooded with fresh, pressurized air to clean the atmosphere (air exchange), resulting in an accelerated decay of particle number (disturbed system). **B**. Contour plot illustrating the temporal variation of particle size distribution (measuring range from 5.6 to 560 nm) in the 22 sec scenario shown in part A. Number concentration ≤ 1E4 pt/ccm is indicated in blue (baseline). Particle size is expressed as electrophoretic mobility diameter. Measurements were performed with a 1 sec resolution.

The exposure chamber atmosphere was characterized by a set of state of the art aerosol monitors, two diffusion – charging instruments (FMPS, miniDISC) and a light – scattering device (CPC). The temporal profiles of total aerosol number concentration, measured either by FMPS or miniDISC, were found to match well for 22 sec particle production, but disagreed progressively with prolonged reactor operation (45 sec, 90 sec) (Figure 1A). Following FMPS, transient peak levels in the range of 1e8 particles per cm^3^ (ccm) were reached during ZnO synthesis. After flame extinction, airborne particle number decreased in an exponential manner by approximately two orders of magnitude over the 30 min exposure time. Comparable time curves were also measured by the CPC (data not shown). Size distribution as analyzed by FMPS (Figure 1B and Additional file 1: Figure S1) revealed a fast increasing particle size (diameter) during and a certain time after reactor operation. Over time, this coagulation processes led to an aerosol with relatively stable size distribution in the range of several 100 nm. With prolonged ZnO production, the size distribution became slightly broader and maximum levels were reached for a longer time, as progressively more particles were produced (in agreement with Figure 1A). Aerosol data were interpreted qualitatively, as the underlying measurements were carried out once. To prove reproducibility, three consecutive runs with 90 sec particle production were characterized. Size distribution - as well as number concentration - profiles were found to be well comparable (data not shown).

### Dosimetry: aerosol deposition measurement

**Figure 2:**
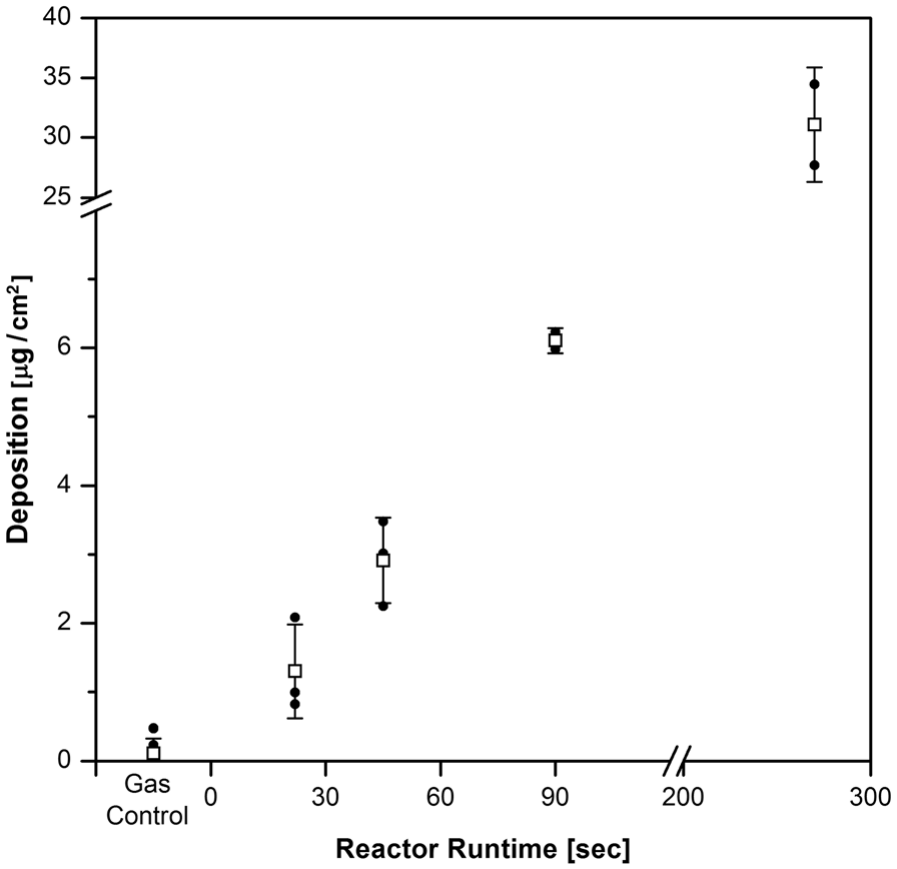
**Aerosol Deposition measurement.** ZnO was quantified by atomic absorption spectroscopy, taking advantage of the solubility of zinc in acetic acid. A ZnO mass deposition of 1.3 – 6.1 μg/cm^2^ was measured in the range of 22 – 90 sec reactor runtime, used for subsequent biological experiments. Points indicate individual measurements, bars and whiskers show mean values and standard deviation. Please note the interrupted axis.

To correlate reactor runtime with a defined dose, the ZnO deposition per area over a 30 min exposure period was determined by element analysis (Figure 2). The average zinc mass per cm^2^ was measured as 1.3 (SD 0.7), 2.9 (SD 0.6), 6.1 (SD 0.2, n = 2) and 31.1 (SD 4.8) μg, in scenarios with 22, 45, 90 and 270 sec reactor runtime. The reactor off – gas control indicated with 0.1 μg/cm^2^ (SD 0.2, n = 6) a clean chamber environment.

### Cytotoxicity

**Figure 3:**
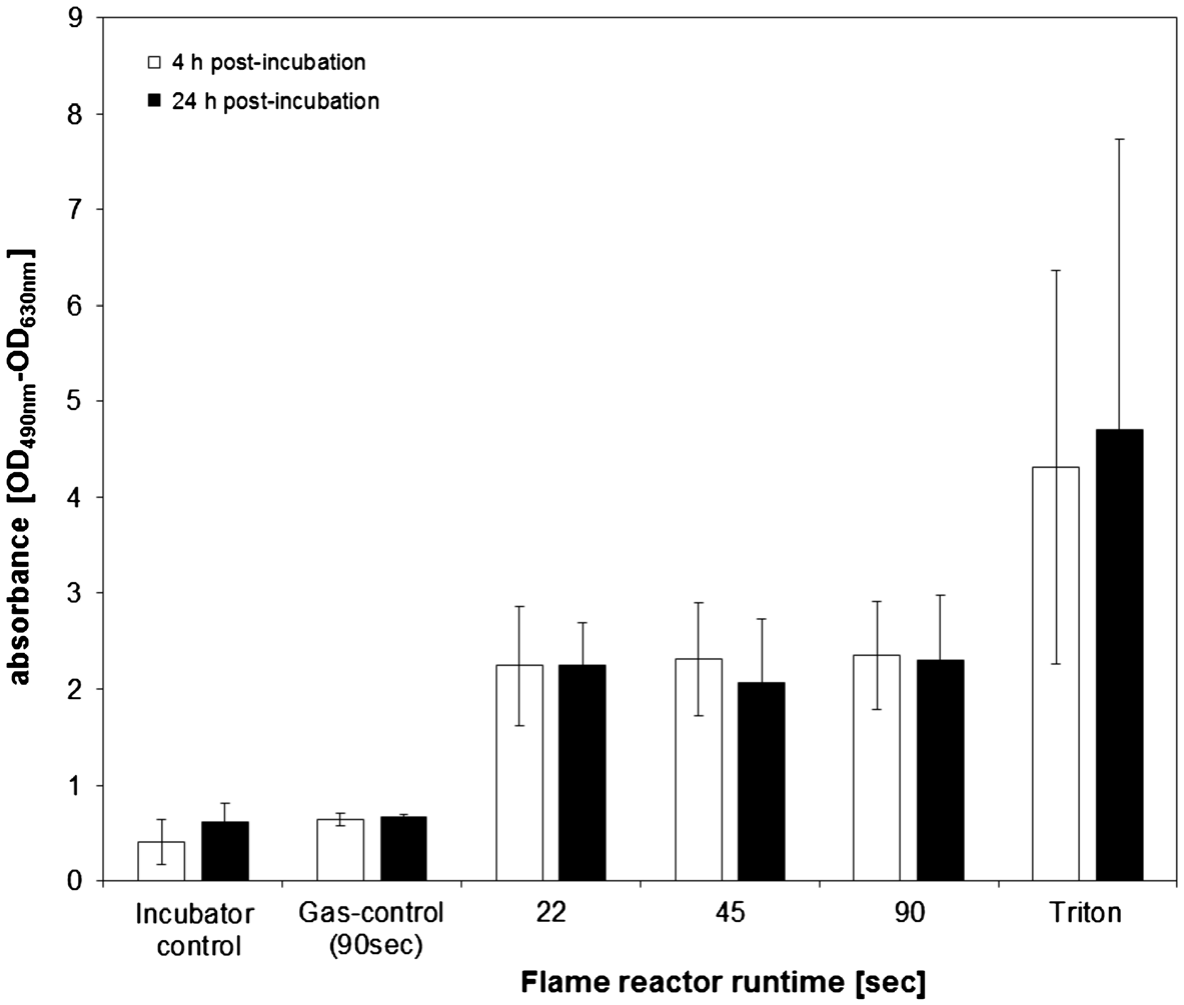
**Cytotoxicity assessed by measuring the release of intracellular lactate dehydrogenase in aerosol exposed samples.** A cell lysate (“Triton”) served as positive control. No difference between incubator – and gas – control could be observed, indicating no influence of the exposure system on basal cytotoxicity. For all ZnO concentrations, elevated LDH levels were measured.

The release of the intracellular enzyme lactate dehydrogenase (LDH) is considered a reliable measure for membrane permeability, and thus an indicator for cytotoxicity (Figure 3). With an optical density (OD) of 0.41 (SD 0.24, n = 6) following 4 h and 0.61 (SD 0.20, n = 6) following 24 h post incubation, the incubator controls shows an LDH release comparable to the gas controls, where ODs of 0.64 (SD 0.07) and 0.67 (SD 0.02) were measured *(p > 0.05)*. These findings illustrate that there is no significant influence of the glove box atmosphere and cell handling on baseline cellular viability levels. Compared with both controls, the extracellular LDH concentrations for particle exposed cell cultures appeared elevated over the entire dose - range for both 4 and 24h post – incubation, an observation which could not be confirmed by the Kruska – Wallis test. An OD of 2.25 (SD 0.62), 2.31 (SD 0.59) and 2.35 (SD 0.56) was measured for scenarios with 22, 45 and 90 sec particle production, following a 4 h period. After 24 h post – incubation, the comparable values were 2.24 (SD 0.45), 2.06 (SD 0.67) and 2.30 (SD 0.68). Both timepoints do not differ significantly *(p>0.05)*. Lysed cell cultures served as positive controls and exhibited values of 4.31 (SD 2.05, n = 4) and 4.71 (SD 3.02, n = 4) for 4 and 24 h incubation time.

### Cellular antioxidant capacity

**Figure 4:**
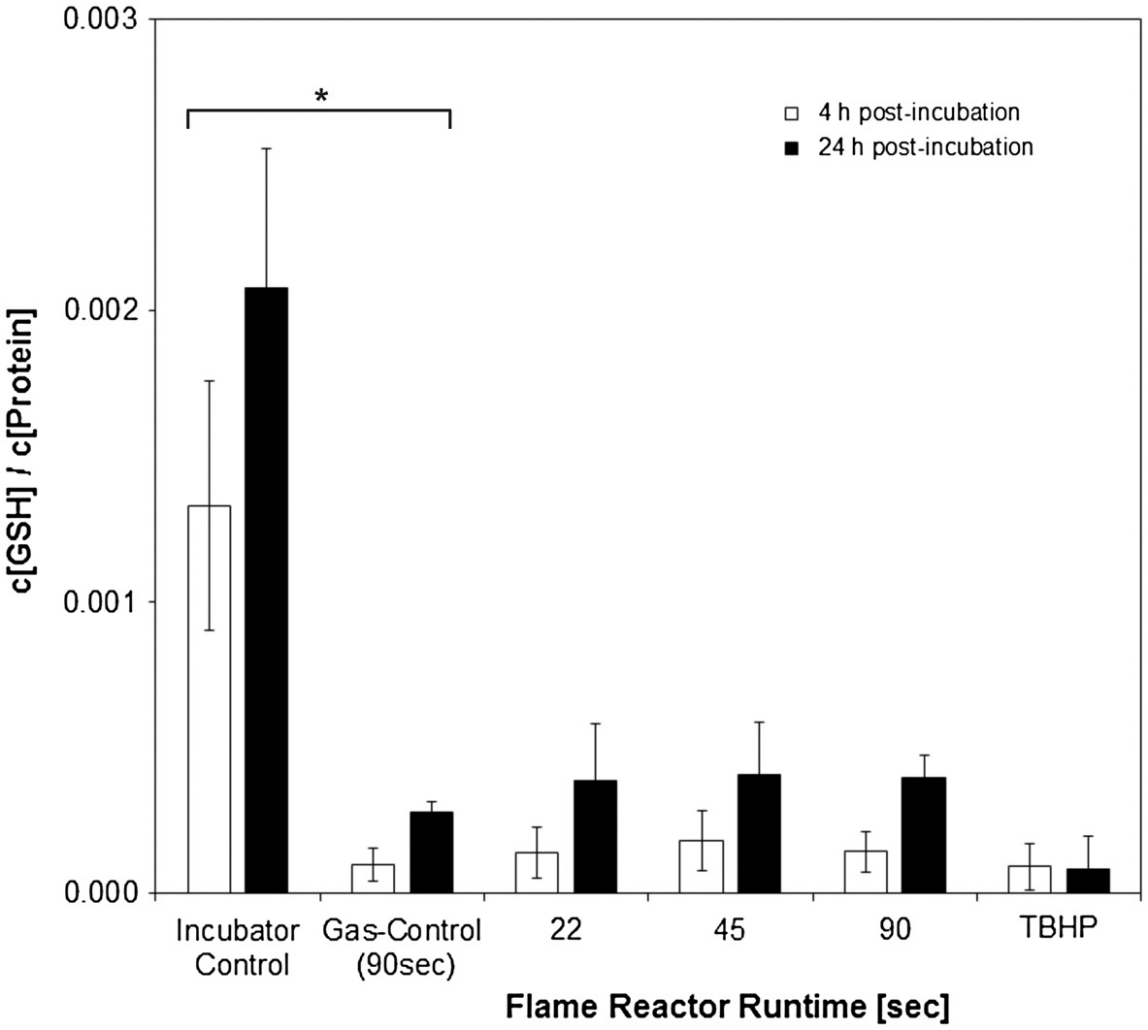
**Quantification of total reduced Glutathione content in aerosol exposed samples.** A comparable reduction was observed for flame off – gases and particle aerosols. *Tert*-Butyl hydroperoxide (TBHP) served as positive control.

The cellular total glutathione (GSH) content (normalized to cellular protein) provides an indirect estimator for oxidative stress. As illustrated in Figure 4, total reduced GSH decreased independent of if cell cultures were either exposed to reactor off-gas (gas – control) or particle aerosols. In the incubator – controls, a GSH to protein ratio of 1.33*10^-4^ (SD 4.30*10^-4^, n = 8) and 2.08*10^-3^ (SD 4.78*10^-4^, n = 8) was measured after 4 and 24 h post – incubation. Gas – controls exhibited ratios of 9.91*10^-5^ (SD 5.81*10^-5^) and 2.79*10^-4^ (SD 3.60*10^-5^). This gas – dependent reduction was found to be statistically significant (both *p = 0.014)* for the 4 and 24 h timepoint. ZnO aerosols produced for 22, 45 and 90 sec led to values of 1.42*10^-4^ (SD 8.84*10^-5^), 1.81*10^-4^ (SD 1.05*10^-4^) and 1.45*10^-4^ (SD 7.09*10^-5^) for 4 h and 3.87*10^-4^ (SD 1.98*10^-4^), 4.11*10^-4^ (SD 1.79*10^-4^) and 3.99*10^-4^ (SD 7.45*10^-5^) for 24 h post – incubation. No statistical difference between gas – control and the different zinc doses was observed. A significant *(p = 0.000)* difference between 4 and 24 h values was found, indicating a certain recovery of cellular antioxidant capacity after prolonged time. With 9.23*10^-5^ (SD 7.94*10^-5^, n = 7) and 8.20*10^-5^ (SD 1.15*10^-4^, n = 7), a maximal reduction was observed for the TBHP positive controls.

### Oxidative stress

**Figure 5:**
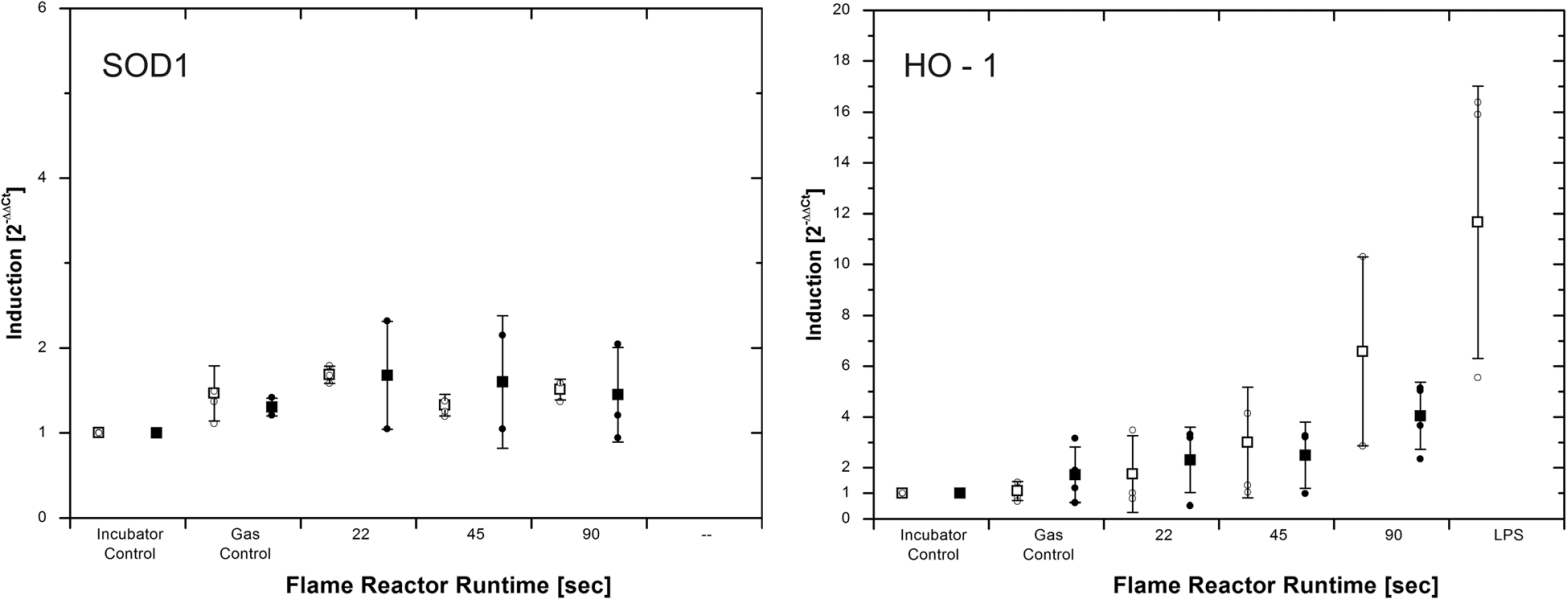
**Real-time PCR on SOD1 and HO-1 in aerosol exposed cultures.** White, open bars represent mean values after 4 h post – incubation, black bars indicate 24 h timepoints. Individual data values are expressed as points. No relevant induction was measured for SOD1. After 4 h, SOD 1 values were 1.5 (SD 0.3), 1.7 (SD 0.1), 1.3 (SD 0.1) and 1.5 (SD 0.1) for Gas – control, 22, 45 and 90 sec particle production. The corresponding data for 24 h were 1.3 (SD 0.1), 1.7 (SD 0.6), 1.6 (SD 0.8), 1.4 (SD0.6). In contrast, HO-1 RNA levels were elevated after 4 and 24 h.

The cellular response to oxidative stress was quantified on the RNA level by doing real-time PCR (RT-PCR) on the antioxidative gene targets superoxide dismutase 1 (SOD1) and heme oxigenase 1 (HO-1) (Figure 5). Compared to the incubator control, no time - or dose – dependent induction was found for SOD1.

A dose – dependent increase of HO-1 transcript was observed but was not significant when applying the Kruska – Wallis test. However, for 4 and 24 h timepoints, a significant (*p = 0.010* and *0.016*) trend and positive correlation with dose (τ = 0.552 and 0.570) was found for HO-1 (Jonckheere – Terpstra - and Kendall’s tau test). After 4 h, HO-1 values were 1.1 (SD 0.4, n = 4), 1.8 (SD 1.5), 3.0 (SD 2.2, n = 4) and 6.6 (SD 3.7) for Gas – control, 22, 45 and 90 sec particle production. The corresponding data for 24 h were 1.7 (SD 1.1, n = 4), 2.3 (SD 1.3), 2.5 (SD 1.3) and 4.0 (SD 1.3). Incubator control and gas control values are comparable for 4 and 24 h post incubation *(p>0.05)*. After 4h, the LPS positive control revealed an induction of 11.7 (SD 5.3), for TNFα, an up-regulation of 8.9 (SD 6.3, n = 4) was observed.

### Release of inflammatory mediators

**Figure 6:**
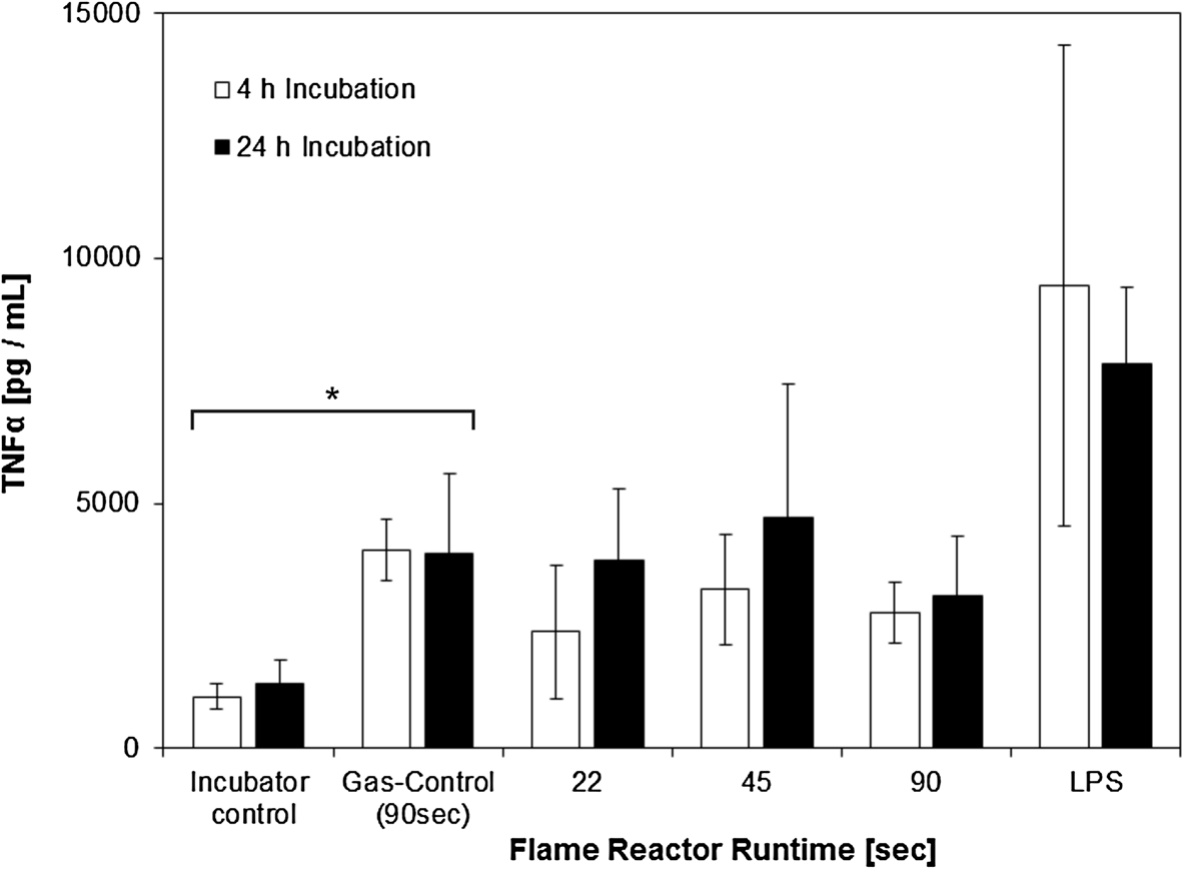
**TNFα release, measured by a BioPlex assay in aerosol exposed cultures.** A significant influence of the gas – atmosphere was found. Lipopolysaccharide (LPS 30 μg/mL) served as positive control.

The potential release of a set of Cytokines (TNFα, IL-1β, IL-6, IL-10,) and Chemokines (IL-8, MIP-1α) into the cell culture medium was quantified by the use of a Multiplex assay. A full overview over the dataset is given in the supplementary information (Additional file 1: Chart S1). IL-8 and IL-6 levels were beyond standard range and were therefore not considered. For MIP-1α, IL-10 and IL-1β, no differences between Incubator (external) control and either gas (internal) control or the different particle doses could be observed following 4 and 24 h post - incubation. In contrast the LPS positive control (30 μg/mL) was elevated for each parameter. Because of its key role in zinc toxicology, and as comparison to data which will be presented later in this manuscript, TNFα values are shown graphically in Figure 6. Incubator and gas control did differ significantly after 4 *(p = 0.024)* and 24 h *(p = 0.036)* post incubation, indicating an influence of flame off - gases on the release of TNFα. Analyzing gas – controls and different zinc concentrations, no influence of dose could be observed (4 and 24 h). Furthermore, TNFα levels 4 and 24 h post – incubations are comparable *(p>0.05)*.

### Suspension scenario

#### Dosimetry: suspension deposition measurement

**Figure 7:**
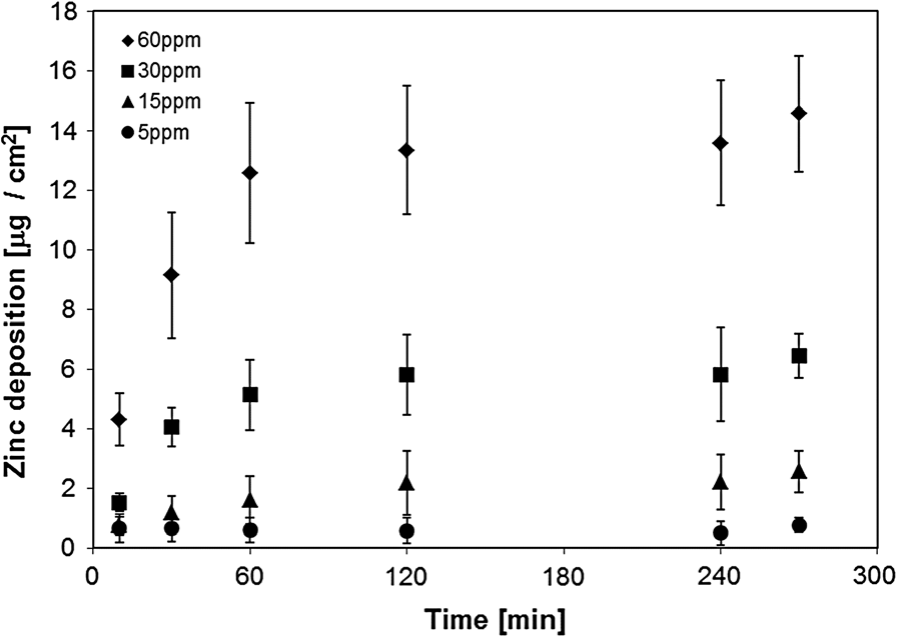
**ZnO deposition onto cell cultures, out of differently concentrated suspensions, measured by atomic absorption spectroscopy.** The deposition reaches a plateau over the first 4 h of incubation. 5 – 60 ppm suspensions correspond to an aerosol – exposure for 22 – 90 seconds. Deposition efficiency increases with concentration. Data are presented as mean ± SD.

Equivalent particle doses are a prerequisite for a comparison of aerosol – and suspension exposure scenarios. The depletion of zinc in the cellular supernatant, ranging from 5–60 ppm (parts per million, = μg/mL culture medium), was assessed over time (Additional file 1: Figure S2), and the obtained data were expressed as mass deposition per culture surface (Figure 7). A plateau was reached over the first two hours, with a mean deposited mass of 0.5 (SD 0.4, n = 4), 2.2 (SD 0.9, n = 4), 5.8 (SD 1.6, n = 4) and 13.6 (SD 2.1, n =4) μg/cm^2^ for 5, 15, 30 and 60 ppm suspensions after 4 h exposure. This is 37.6, 55.8, 73.8 and 86.0% of the maximal possible deposition, which was calculated to 1.3, 3.9, 7.9 and 15.8 μg/cm^2^. A dose range of 5 – 30 ppm was found to be comparable, following 22 – 90 sec particle production (1.3 – 6.1 μg/cm^2^) in the aerosol scenario. Basing on this knowledge, suspension experiments were performed with ZnO suspensions of 0 – 30 ppm, as previously investigated by Brunner *et al.*[13], with additional 60 and 80 ppm suspensions as “high dose” controls.

### Suspension characterization

Suspensions were characterized by dynamic light scattering to get an insight into size distribution and agglomeration state/- kinetics (Additional file 1: Figure S3). The dissolution behavior of the flame – made ZnO particles, used in this study, was assessed as a function of time (Additional file 1: Figure S4). A solubility of about 4% was observed independent of timespan (up to 4 h) and incubation procedure (stirred, calm environment), indicating, that zinc interacts with the cell cultures as particulate ZnO.

### Cytotoxicity

**Figure 8:**
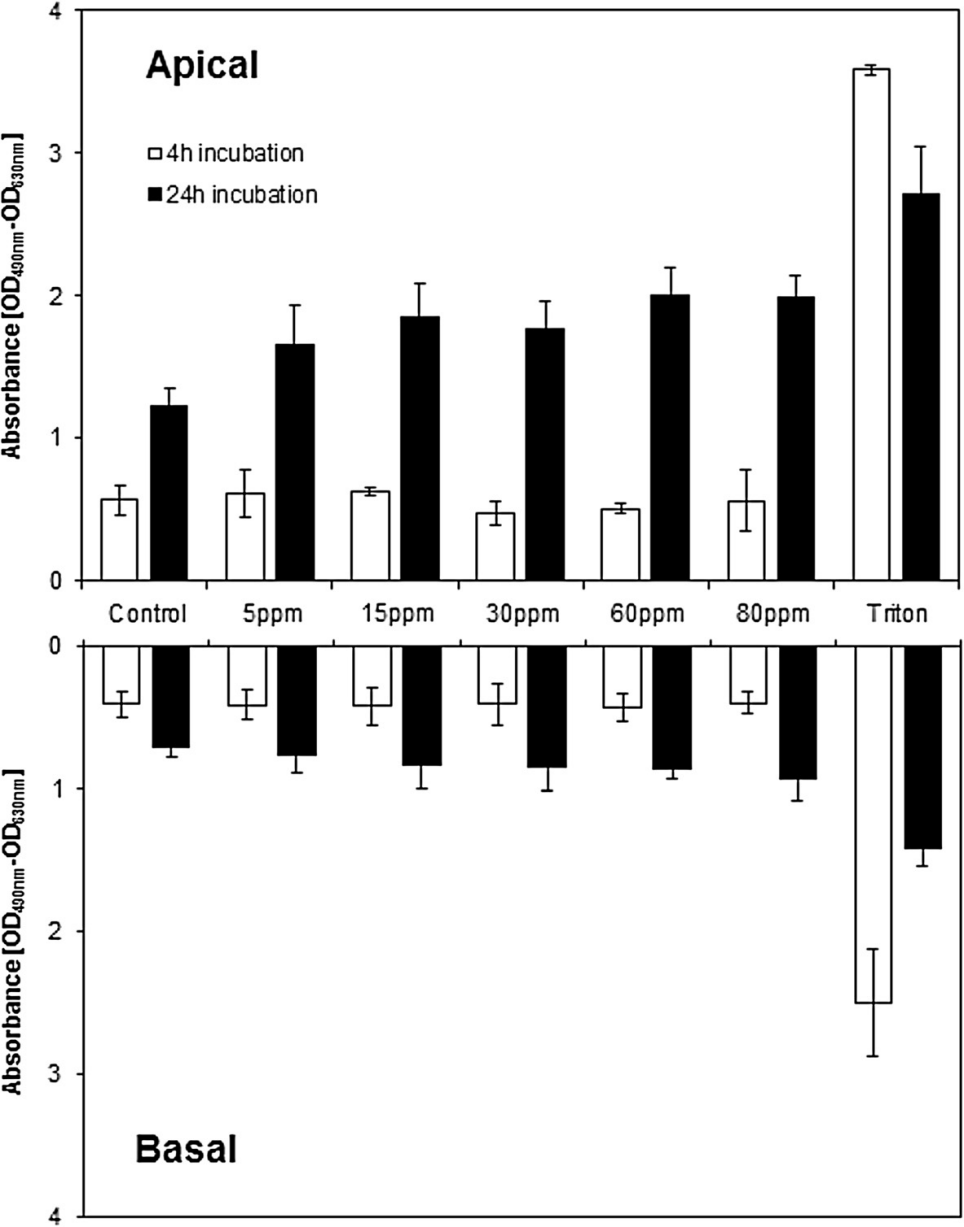
**LDH release measured in the upper and lower well of the cell culture inserts.** Absorption values are expressed as optical density (OD), measured at 490 nm, with 630 nm as reference wavelength. LDH is generally elevated after 24 h incubation time.

Samples were taken from the apical (upper) and the basal (lower) compartment of the triple cell co -culture, to investigate the contribution of the different cell populations to the total LDH release (Figure 8). With an OD of 0.57 (SD 0.10), 0.61 (SD 0.17), 0.62 (SD 0.03), 0.47 (SD 0.08), 0.51 (SD 0.04) and 0.85 (SD 0.21) in the apical, and of 0.41 (SD 0.09), 0.41 (SD 0.11), 0.42 (SD 0.13), 0.41 (SD 0.15), 0.41 (SD 0.06) and 0.40 (SD 0.07) in the basal compartment, the extracellular LDH concentrations remained stable after 4 h exposure to 0, 5, 15, 30, 60 and 80 ppm ZnO.

After 24 h zinc exposure, values of 1.23 (SD 0.12), 1.66 (SD 0.27), 1.85 (SD 0.23), 1.77 (SD 0.20), 2.00 (SD 0.19) and 1.98 (SD 0.16) were measured on the apical - and 0.71 (SD 0.08), 0.77 (SD 0.12), 0.84 (SD 0.16), 0.85 (SD 0.16), 0.86 (SD 0.07) and 0.94 (SD 0.14) on the basal side of the culture insert. A dose - dependent correlation (τ = 0.591 and 0.355) was observed for 24 h exposure in the apical *(p = 0.001)* and basal *(p = 0.050)* compartment by the Kendall’s tau test. However, the Friedman global test did not show any significance. LDH release after 4 and 24 h exposure differed significantly in both compartments *(p = 0.000)*, indicating a more pronounced cytotoxicity after prolonged time.

### Total reduced glutathione capacity

**Figure 9:**
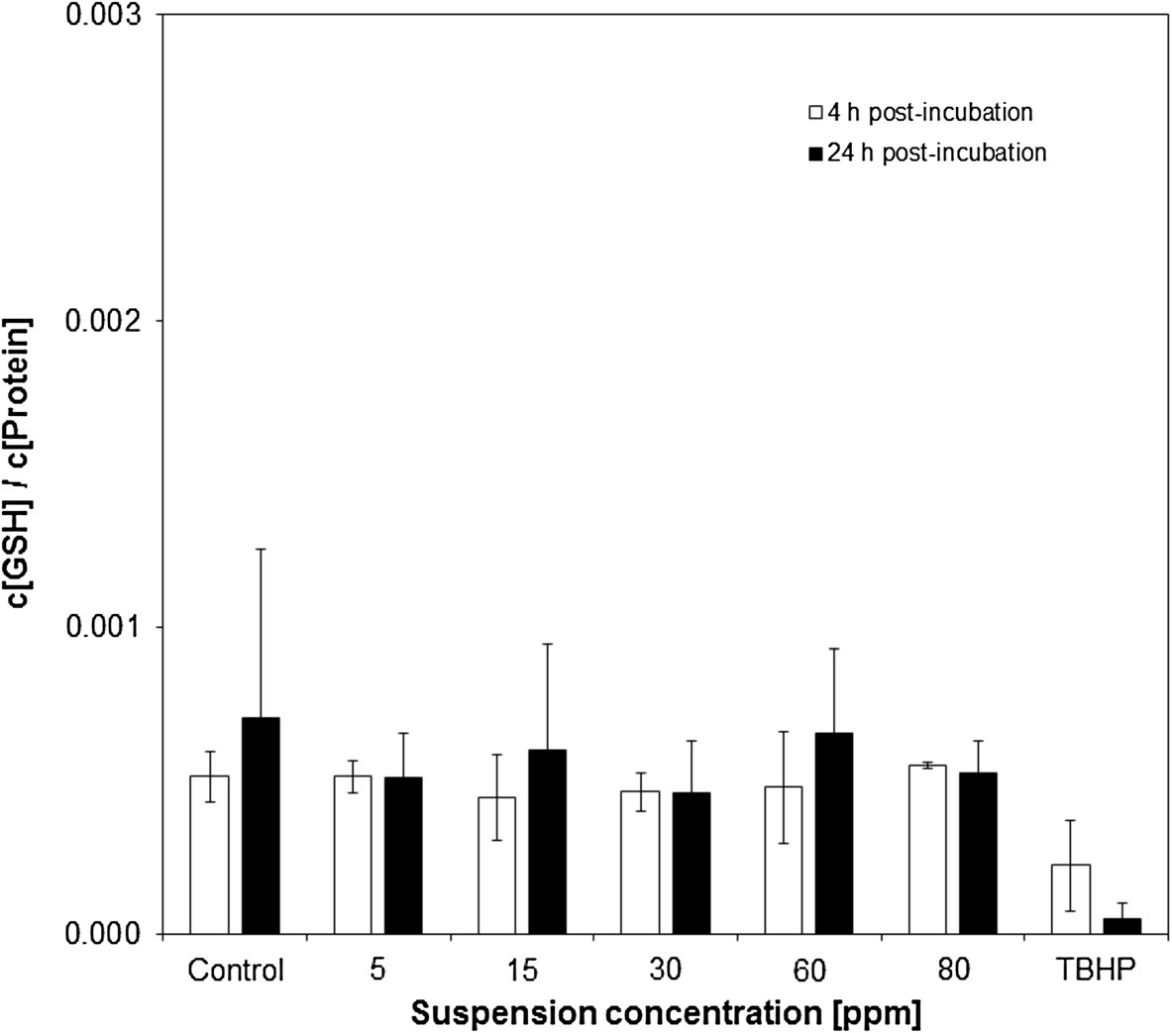
**Total reduced Glutathione content in suspension exposed cultures.** No depletion of GSH could be found.

No regulation of the total reduced GSH was found over the entire dose range after 4 and 24 h exposure time (Figure 9). After 4 h, the GSH/protein ratio was 5.12*10^-4^ (SD 8.07*10^-5^), 5.13*10^-4^ (SD 5.25*10^-5^), 4.42*10^-4^ (SD 1.4*10^-4^), 4.62*10^-4^ (SD 6.34*10^-5^), 4.78*10^-4^ (SD 1.82*10^-4^) and 5.48*10^-4^ (SD 1.03*10^-5^) for 0 to 80 ppm. The corresponding data for 24 h (all n = 5) were 7.04*10^-4^ (SD 5.48*10^-4^), 5.10*10^-4^ (SD 1.41*10^-4^), 6.01*10^-4^ (SD 3.43*10^-4^), 4.60*10^-4^ (SD 1.71*10^-4^), 6.54*10^-4^ (SD 2.75*10^-4^) and 5.22*10^-4^ (SD 1.07*10^-4^).

### Oxidative stress

**Figure 10:**
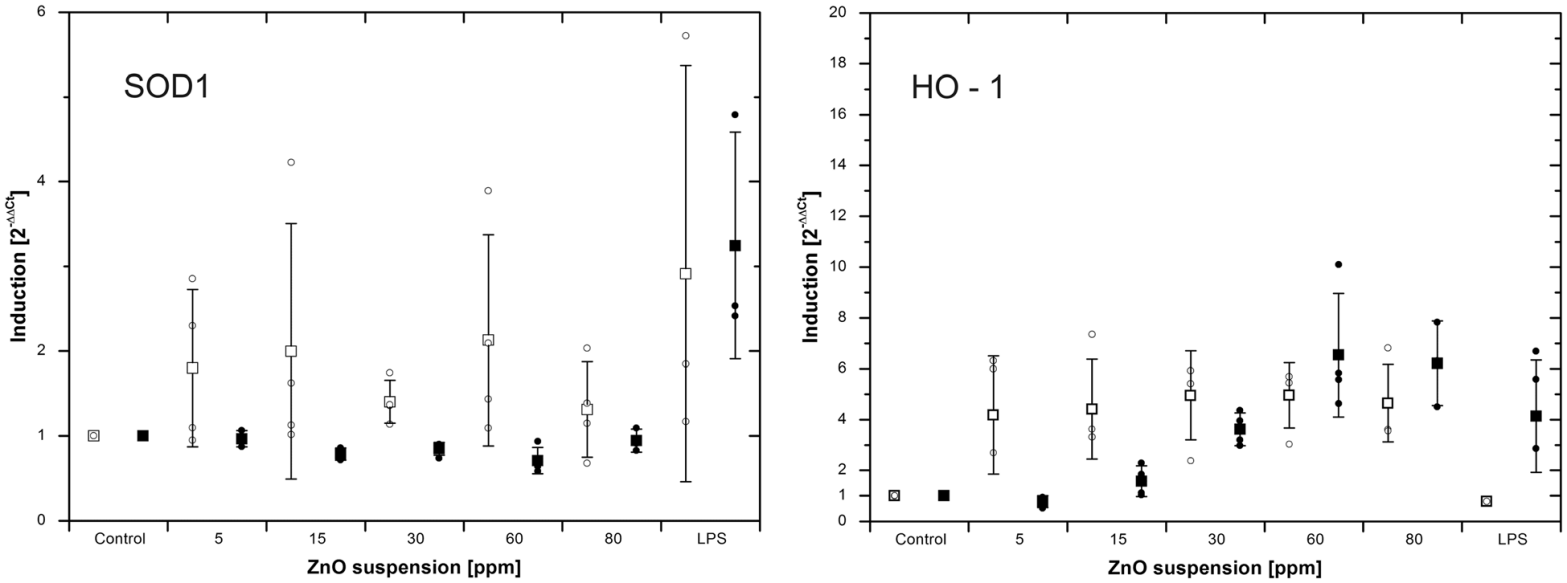
**Real-time PCR on SOD1 and HO-1 in suspension exposed cultures.** No regulation of SOD1 was found. In contrast, HO-1 is constantly induced (over the entire dose range) after 4 h incubation. Furthermore, a dose dependent increase was observed after 24 h. White, open bars represent mean values after 4 h post – incubation, black bars indicate 24 h timepoints. Individual values are expressed as data points.

SOD1 remained on basal level for all conditions and timepoints (Figure 10). After 4 h exposure, HO-1 values were 1.0 (SD 0.0), 4.2 (SD 2.3), 4.4 (SD 2.0), 5.0 (SD 1.7), 5.0 (SD 1.3) and 4.6 (SD 1.5) for concentrations of 0 ppm to 80 ppm (all n = 4). No significant effect was found by the Friedman test *(p>0.05)*. The corresponding data for 24 h were 1.0 (SD 0.0), 0.8 (SD 0.2), 1.6 (SD 0.6), 3.6 (SD 0.6), 6.5 (SD 2.4) and 6.2 (SD 1.7) (all n = 4).). After 24 h exposure, a significant induction was demonstrated by the Friedman test *(p = 0.012)* and a significant dose dependent trend and correlation of dose and HO-1 transcript was found by the Jonckheere – Terpstra – and Kendall’s tau analysis (*p = 0.000,* τ = 0.776). The LPS positive control was determined as 1.8 (SD 1.5) and 6.1 (SD 0.8) for 4 and 24 h (n = 2).

### Release of inflammatory mediators

**Figure 11:**
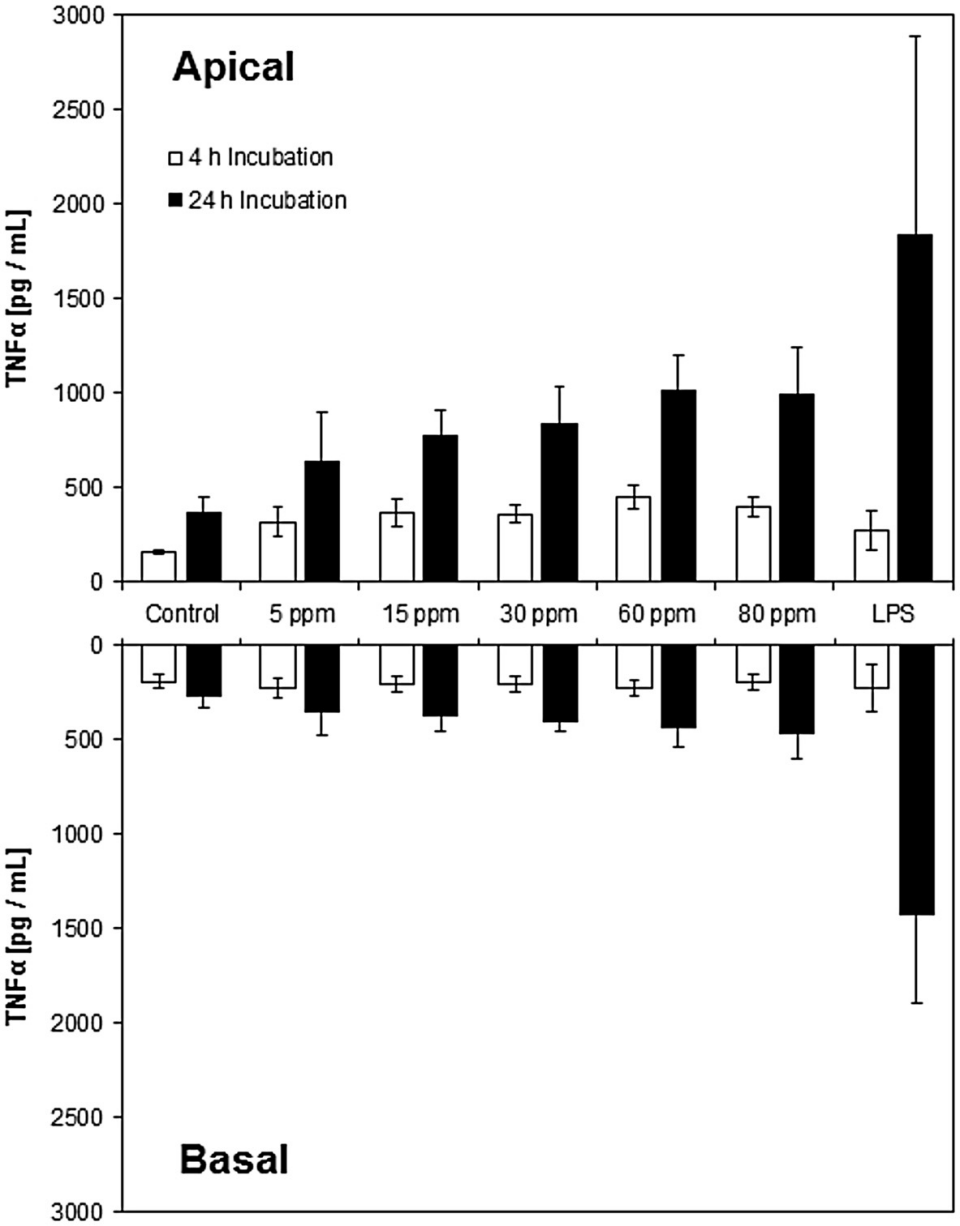
**TNFα release, quantified by a BioPlex assay.** A dose dependent release was found for the apical compartment after 24 h.

The release of inflammatory mediators was assessed by the same 6 - Plex BioPlex assay as for the aerosol scenario (data summary in Additional file 1: Chart S2). TNFα, known in the literature to be up regulated by the presence of zinc, is shown (Figure 11). In the apical compartment, a significant effect of ZnO after 4 and 24 h exposure was demonstrated by a Friedman test (*p = 0.01*5 and *0.035*) and also a significant (*p = 0.003* and *0.001*) dose dependent trend and positive correlation (τ = 0.536 and 0.619) was found by Jonckheere – Terpstra – and Kendall’s tau analysis. On the basal side, a significant effect was only observed at the 24 h timepoint by Friedman analysis *(p = 0.048)* and Jonckheere – Terpstra – and Kendall’s tau test (*p = 0.005*, τ = 0.508). Additionally, a significant difference was observed for both exposure times in the apical and basal compartment *(p = 0.000)*.

## Discussion

The aim of this study was to assess the toxicology of aerosolized ZnO when exposed to a realistic triple cell co – culture model of the human epithelial airway barrier, and to provide a comparison with a dose – equivalent suspension scenario. A large number of *in vitro* models of the histologically different pulmonary epithelial barriers are currently available, and many have already been used to investigate particle – lung interactions. Traditionally, experiments have mainly been performed using monocultures of single cell lines or isolated primary cells [47]. Such monocultures can be taken as “simplistic” models of a tissue of interest such, in this case the lung. In reality, biology is much more complex. The airway barrier is a multidimensional structure and consists of many different specialized cell types, including epithelia, macrophages and dendritic cells among others [48], wherefore increasing emphasis was put on the development of sophisticated 3D co - culture models. Against this background, an advanced, well established and characterized triple – cell *in vitro* model of the human respiratory tract, whose focus lies on cellular crosstalk, was chosen for this study [46,49,50]. In order to simulate aerosol – inhalation as close as possible, the here used *in vitro* exposure system combines realistic particle production and defined aerosol aging (in terms of *i. a.* agglomeration) with a dose – controlled, passive mass deposition by means of diffusion and sedimentation. The combination of a sophisticated exposure system with a realistic cell culture model of the airway epithelium provides us with a reliable tool to investigate aerosol toxicology under “quasi – realistic” occupational conditions.

Nowadays, studies on workplace related exposure to airborne submicron particles are numerous [5,8]. Investigations on particle emissions from pilot scale flame – reactors, comparable to the one used in this study, revealed peak number concentrations in the range of 10^4^ to 10^5^ pt/ccm during regular particle production, what was about an order of magnitude above background concentrations [9,11]. These data represent routine working conditions. In hazardous situations however (neglected or missing worker health protection, leaks etc.), a substantial higher amount of particle may be released unintentionally into the environment during particle manufacture. Such a situation may occur especially within industrial settings, as simulated in this study, where huge quantities of particulate matter are being produced and handled. Deliberated particles undergo various modifications over time, as they are subject to aging processes like agglomeration, dissolution of the stabilizing shell and full or partial dissolution of the nanoparticle [14]. Agglomeration is a spontaneous and ever – present interparticle phenomenon for aerosols, resulting in a continuous decrease of number concentration, coupled with an increase in particle (agglomerate) size [44], a pattern demonstrated for the ZnO aerosols used in this study (Figure 1B and Additional file 1: Figure S1). The obtained aerosol data were in good agreement with predictions made for monodisperse coagulation, such as a reduction of aerosol number concentration of approximately two orders of magnitude over an exposure time of 30 min for about 10^8^ pt/ccm initial concentration [3,44].

The deposited ZnO dose (mass per surface area) of 1.3 – 6.1 μg/cm^2^ measured in this study was found to be comparable with a previous ZnO aerosol – and suspension exposure study carried out by Lenz *et al.*[21], where dose levels of 0.3 – 8.5 μg/cm^2^ were obtained for the aerosol scenario using an air-liquid exposure system by nebulizing a particle suspension over the surface of lung cells. To put the dose into perspective, it is important to consider, that these doses are within the maximum alveolar lifetime dose accumulated by a worker, which was calculated as 3.6 – 18 μg/cm^2^, based upon lung morphology, physiology and recommendations of the Occupational Safety and Health Administration (OSHA) (5 mg/m^3^ threshold value) [21].

Dose – equivalence is a prerequisite for a comparison of aerosol – and suspension exposure scenarios. Under submerged conditions, the Zinc depletion of the cell culture’s supernatant was taken as indirect measure for particle deposition. It could be shown by AAS analysis that most of the administered zinc (on a mass base) has reached the cells after a 2 h exposure period (Figure 7). This fast particle transport can be explained by the high agglomeration tendency of ZnO particles, when dispersed in cell culture medium or water (non – stabilized suspensions) [17] (Additional file 1: Figure S3). Over time, large clusters are formed which are deposited with high efficiency (large mass) due to gravitational settling (sedimentation) [17].

Beside the administration of identical amounts of ZnO, it is also essential that suspended as well as aerosolized particles reach the cells in a comparable sate of agglomeration. However, the measured size distributions are generally difficult to compare, as the specific measuring principles of the different particle monitors differ fundamentally. However, the output metrics (mobility equivalent diameter in the given matrix), allow, from a pragmatic point of view, an (at least qualitative) correlation of the particle size data determined by FMPS and DLS.

At first sight, aerosol and suspension data seems to differ significantly, especially with regard to the maximal particle size over prolonged time, which appears much higher in the suspension scenario (Additional file 1: Figure S3). Considering the immanent limitations of FMPS such as the systematic underestimation of fractal particle size and the limited measuring range, the “real” particle diameter seems to far exceed the measuring range (560 nm) already soon after the extinction of the flame due to fast agglomeration (a result of the generally high particle mobility in the gas phase, the high aerosol concentration and the actively mixed atmosphere). Thus, Figure 1 and Additional file 1: Figure S1 show an adequate picture of the initial phase of the experiment (particle production, initial agglomeration) but the FMPS massively underestimate particle size over more prolonged time. In view of the above considerations, the particles size range in either gas or liquid seems to saturate in a comparable order of magnitude (micron range), thus excluding biological effects arising solely from differences in agglomeration state/particle size between the two exposure scenarios.

There is evidence in the literature, that the toxicity of ZnO is mainly driven by the extracellular release of Zn(II) – Ions followed by their subsequent uptake into cells most likely through zinc transporters. Additionally, several ZnO particle - preparations were found to be highly soluble in cell culture medium [51]. To clarify the amount of dissolved zinc in our experimental system, the solubility (in cell culture medium) of the flame – made ZnO particles, used in this study, was analyzed (Additional file 1: Figure S4). Independent of timespan (up to 4 h) and incubation procedure, only about 4% of the initial ZnO dose (≥ as the maximal added dose in a biological experiment) was dissolved. This reflects the improved stability of the here used ZnO as it has been prepared with the corresponding industrially relevant high temperature process (see experimental section). A number of earlier reports on ZnO used nanoparticles from low temperature synthesis, later are not sintered and dissolve rapidly. In this study, we deliberately chose the industrially more relevant material, as to provide a test material as close as possible to eventual human exposure scenarios [13,15]. This finding demonstrates that zinc reaches the cells mainly as ZnO particles and not in an ionic state. This mechanism has been named a Trojan horse type uptake and was first experimentally observed using similar, industrially relevant ZnO nanoparticles (Brunner *et al.*[13]) and later confirmed by Xia *et al.*[30].

A dose - dependent reduction of cell viability is described in the literature following contact of ZnO particles with cell lines derived from human lung (BEAS-2B, A549) [31,36], nasal mucosa [37], aorta endothelium [38], the kidney [32] and the lymphatic system [33]. Additionally, increased cytotoxicity (reduced viability) was measured directly in BEAS-2B, RAW264.7 macrophage, MSTO-211H mesothelioma and 3T3 rodent fibroblast cell lines [13,30]. In line with these findings, a pronounced ZnO - cytotoxicity was observed in the 16HBE14o- triple cell co-culture model under aerosol and suspension exposure conditions, as demonstrated by measuring extracellular lactate dehydrogenase (LDH) activity.

Compared to either incubator - or gas – control, all aerosol concentrations induced an elevated, but statistically not significant, LDH release for 4 and 24 h timepoints (Figure 3), indicating, that the dose – response curve seems to reach its plateau already at or before a dose, corresponding to 22 sec particle production. Compared to the Triton positive – control, the values appears as to be comparably high. As a maximal cytotoxicity was already reached after 4 h, and no further cell death could be detected over prolonged time (no time – course of acute toxicity), a certain cell population of the cultures remains viable under all conditions. It can be hypothesized that the different cell types that the co – culture is composed of (epithelial cells, macrophages, dendritic cells) may differ in their susceptibility to zinc, and that the most responsive cell category would be eradicated first, followed by the other populations ordered along a gradient of their susceptibility to ZnO. In addition, ZnO may be deposited on local “hotspots”, where the particle or agglomerate define a radius within which cells may be subject to focal toxicity. Cells localized outside of this area would be protected of any adverse effects and therefore remain viable. In the suspension scenario, no LDH release was observed after 4 hours on the apical and basal side. However, after 24 h post – incubation the values appeared significantly increased in both compartments.

The immanent ability of particles to induced oxidative stress, a phenomenon resulting from an imbalance in the cellular oxidant - antioxidant equilibrium in favor of the oxidants, is a fundamental mechanistic paradigm in (nano) toxicology. Particles may cause oxidative stress by triggering excessive (biotic or abiotic) generation of reactive oxygen species (ROS) so that the cellular antioxidant defence mechanisms may be overwhelmed by these oxidants [52,53]. The ability of ZnO to cause elevated ROS levels was demonstrated in several studies e.g. for RAW264.7 mouse macrophage and BEAS-2B human alveolar epithelial cell lines [30], A549 human bronchioalveolar cells [31], human kidney cell lines (IP15, HK-2) [32], a mouse macrophage line (Ana-1) [54] and human lymphoplastoid cells (WIL2-NS) [33]. Furthermore, cell – free (“abiotic”) ROS generation was identified as a material – specific property of ZnO [30,54]. Under physiological conditions, the steady-state formation of cellular oxidants, an attribute of aerobic life, is balanced by a similar rate of their neutralization by antioxidants that are of enzymatic and non-enzymatic origin [52]. In contrast, oxidative stress causes a depletion of protective cellular antioxidants due to overburdened defence mechanisms. In this context, the essential, ubiquitous non-enzymatic radical scavenger glutathione (GSH) [55] is of great importance and its depletion can be used as indirect measure for oxidative stress. Reduced cellular GSH concentrations upon ZnO exposure were already measured in several studies [31,32].

The quantification of total reduced glutathione in aerosol – samples revealed a distinct loss of total reduced GSH when cells were either exposed to flame off-gases or to all different particle concentrations at any timepoint (Figure 4). This behavior indicates a strong effect of the gaseous components of the aerosol on the degree of cellular oxidative stress. Interestingly, also after removal of the factor “flame – gases” and 24 h post – incubation, the values do not recover to baseline. This may be evidence of the fact that the cells undergo persistent and irreversible alterations by the gas components. It is also conceivable, that the initial reduction of total GSH at 4 h was caused by gases, an effect which was later on (after 24 h) overtaken by the deposited zinc particles.

In the suspension scenario, no alteration of total GSH was observed under any conditions (Figure 9). This finding supports the assumption that interaction of cell cultures at the air – liquid interface with the gas atmosphere may strongly influence the toxicological outcome. The gas components of an aerosol are adding an additional layer of complexity to this scenario, whose influence needs to be assessed for each individual factor. As combustion by product of flame spray pyrolysis, gaseous compounds such as CO_2_, CO, NO_x_, are being released [22]. It was shown in the literature that exposure to filtered (particle free) diesel exhaust induces an acute reduction of the cellular GSH content, whereby glutathione levels were restored to control levels 24 h after a 1 h exposure [56]. This finding is in contrast to the current study, as the reduction of intracellular glutathione was not reversible by placing the cultures for 24 h in an incubator. A prominent nitric oxide, namely the oxidant NO_2_, was found to directly interact with GSH, causing a reduction of glutathione [57]. This proves a mechanistic link between nitric oxide and the cellular antioxidant capacity.

In order to organize cellular responses to particles along a gradient of oxidative stress, a “hierarchical oxidative stress” model has been proposed [30,53]. Following this well-established paradigm, the lowest level of oxidative stress (Tier 1) is associated with the induction of a battery of antioxidant defence genes by redox – sensitive transcription factors, mainly Nrf2 [58], to restore redox homeostasis. A characteristic marker among this panel is HO-1, an antioxidant enzyme which was found to be up - regulated upon exposure to diesel exhaust [34], the water insoluble fraction of fly ash [59], cerium oxide [60] and ZnO [21,30]. We have found that the pattern of HO-1 regulation differed among the two exposure scenarios. In aerosol exposed samples, gene induction correlates positively with applied dose for 4 and 24 h (Figure 5). In the suspension scenario, HO-1 appears elevated for 4 h and shows a significant dose – dependent trend for 24 h (Figure 10). Incubator and gas – control do not differ significantly, indicating that gas components alone are not able to induce heme oxygenase directly, although gas exposure causes oxidative stress as demonstrated by the reduction of intracellular reduced GSH. Consistently, also no indication for any oxidative stress can be derived from the GSH data in the suspension scenario, however a regulation of HO-1 was observed. The HO-1 induction is shaped mainly by ZnO exposure.

A further important line of antioxidant enzyme defence is the superoxide dismutase family (SOD), whereof, the SOD1 isoform (CuZn-SOD) was chosen as additional target in this study. In both scenarios, no regulation of SOD1 was observed (Figures 5 and 10).

Further escalation of oxidative stress (Tier 2 level) is supposed to induce a number of pro - inflammatory pathways, such as mitogen – activated protein kinase (MAPK) - and NFκB cascades, resulting in the release of a wide array of cytokines and chemokines. Although the underlying regulatory mechanisms are little researched, ZnO was found to trigger the *in vitro* translation of inflammatory mediators such as TNFα [30,35] and IL-8 [30,36] in several cell culture systems. For the latter, also transcriptional activation was observed [21,38]. Furthermore, a number of studies clearly demonstrated elevation of a set of inflammatory biomarkers (TNFα, Il-8, Il-6, IL-1β) in lung lavage fluid of human volunteers upon inhalation exposure of ZnO aerosols (“fumes”) at high concentrations. ZnO dose was found to correlate with TNFα, IL-8 and IL-6 response for several timepoints [61,62]. The observed temporal pattern lead to the hypothesis, that the early increased TNFα plays an important initial role in the pathology of metal fume fever, as it may lead the secondary release of IL-8 and IL-6 in the pulmonary environment.

To assess the pro – inflammatory potential of ZnO, a set of inflammatory mediators such as TNFα, IL – 8, IL – 6 and IL - 1β was assessed in this context. Only TNFα concentrations were found to be affected by zinc oxide. In the aerosol scenario, TNFα levels remained on gas – control baseline after 4 and 24 h (Figure 6, Additional file 1: Chart S1). However, gas – and incubator – control differed significantly, illustrating, that the basal TNFα level is elevated in glove box exposed cultures as a result of gas exposure. In the suspension scenario, a significant time and dose dependent increase of the extracellular TNFα concentration was observed for both timepoints in the apical, ZnO exposed, compartment. After 24 h, such an effect was also found in the basal well.

## Conclusion

An aerosol is a complex mixture of gaseous and particulate components which may both trigger adverse health effects. This characteristic is of special importance in the case of combustion derived particles, as high – temperature processes such as flame synthesis release chemically active gases as by products. In the current study, flame off gas was found to elicit a significant decrease of total reduced GSH and to induce further the release of the pro – inflammatory cytokine TNFα. Such findings demonstrate the contribution of the gas phase on aerosol toxicology. Removal of the factor “flame gases” by performing suspension exposure allows distinguishing particle derived effects from the (masking) influence of gaseous compounds. Under this constellation, the total reduced GSH remained stable over the investigated dose range and TNFα levels correlated positively with ZnO – dose. Other parameters such as LDH and HO-1 were not directly influenced by flame off gases and showed particle associated effects in both scenarios: Following aerosol exposure, LDH levels appeared elevated at both timepoints and HO-1 transcript correlated positively with deposited zinc dose after 4 h. Under submerged conditions, the HO-1 induction scheme deviated for 4 and 24 h and increased extracellular LDH could be found only following 24 h exposure. From our results it can be concluded that both exposure strategies differ fundamentally in their dose - response pattern. Additional differences can also be found for the factor time: In the aerosol scenario, the investigated parameters tend to their maximum already after 4 h of exposure, whereas under submerged conditions, effects appear most pronounced mainly after a 24 h incubation period.

If different exposure scenarios are compared the same particles have to be used and thoroughly characterized. Additionally, the dose of particles reaching the surface of the cells and how much is taken up the cells is another requirement for a direct comparison. In accordance with our findings it can be concluded from an occupational health perspective that the adverse potential of a flame made ZnO aerosol is determined to a considerable extend by the effects of flame off gases, which act in concert with particle specific responses.

## Methods

### Aerosol exposure system

Technical parameters and specifications of the exposure chamber have previously been published elsewhere [15,22,42,43,63]. In principle, the system combines particle manufacturing and aerosol-exposure of lung cells within one spatial compartment. This is achieved with a well stirred custom made glove-box apparatus which encloses all required infrastructure to expose cell cultures, together with the nozzle of a pilot scale aerosol reactor. For the here reported experiments, ZnO aerosols were generated by flame spray pyrolysis [64], using zinc 2-ethylhexanoic acid diluted in xylene as precursor [65]. The liquid was fed at a rate of 5 mL min^-1^ (capillary diameter 0.4 mm) into a methan-oxygen supporting flame (oxygen flow 5 L min^-1^).

### Aerosol deposition measurement

BD Falcon™ cell culture inserts (surface area 4.2 cm^2^; Becton Dickinson, Allschwil, Switzerland) were placed in 6-well BD Falcon™ tissue culture plates (Becton Dickinson, Allschwil, Switzerland) and filled with ~ 1 mL 0.5 M acetic acid. The membrane inserts were exposed to the glove box atmosphere and the ZnO reaching the membrane inserts was dissolved. For chemical zinc quantification, the samples were further diluted (0.5 M acetic acid) to the working range (< 2 ppm) of atomic absorption spectroscopy (AAS). Measurements were carried out by the use of a SpectrAA 220FS Atomic Absorption Spectrometer (Varian Inc., Palo Alto (CA), USA) with the following instrument settings: Flame type: Air/Acetylene, Flow: 13.5/2.0 L min^-1^, Burner Height: 13.5 mm, Wavelength: 213.9 nm, Slit width: 1.0 nm, Lamp current: 5.0 mA. All other AAS measurements mentioned in this manuscript were carried out according to this protocol.

### Aerosol characterization

Aerosol was sampled near the location of cell cultures and was diluted by a rotating disc diluter (Model MD 19 – 2E, Matter - Aerosol AG, Wohlen, Switzerland) operating at potentiometer setting 1.0 (dilution 1:118). The conductive tubing connecting glove box and diluter was ~ 1.5 m long, with 5 mm inner diameter. A condensation particle counter model CPC 3007 (TSI GmbH, Aachen, Germany) and a portable diffusion size classifier DiSCmini SN25 [66] (University of Applied Sciences and Arts Northwestern Switzerland, Windisch, Switzerland) were connected directly to the diluter head. A further flow branch was diluted a second time with a clean air flow of ~5.9 l/min to run/operate a Fast Mobility Particle Sizer (FMPS), Model 3091 (TSI GmbH, Aachen, Germany). The aerosol measuring devices were all calibrated recently and flows were checked prior to measurement with a Bios defender flowmeter.

### Triple cell co-culture model

Exposure experiments were carried out with a triple cell co-culture model of the human epithelial airway barrier, previously described in detail in [45,46,49]. Briefly, monocultures of the human bronchial epithelial cell line 16HBE14o- were cultivated in Minimal Essential Medium (MEM with Earle’s Salts, w/o L-Glutamine, Gibco BRL Life Technologies Invitrogen AG, Basel, Switzerland), supplemented with 1% penicillin G/streptomycin sulfate (10,000 units mL^-1^/10,000 μg mL^-1^, Gibco B-RL, Invitrogen AG, Basel, Switzerland), 1% L-Glutamine (LabForce AG, Nunningen, Switzerland) and 10% fetal bovine serum (LabForce AG, Nunningen, Switzerland), subsequently referred to as “MEM complete medium”. For exposure experiments, cells were seeded in BD Falcon™ cell culture inserts (surface area 4.2 cm^2^; Becton Dickinson, Allschwil, Switzerland) placed in in 6-well BD Falcon™ tissue culture plates (Becton Dickinson, Allschwil, Switzerland) at a density of 0.5 × 10^6^ (aerosol scenario) or 1 × 10^6^ cells/insert (submerged scenario). After 5 days (6 days for the submerged scenario respectively), the co-cultures were put together by adding 500 μL of a human monocyte - derived macrophage suspension to the apical – and 300 μL of human monocyte derived dendritic cells to the basal side of the insert. Monocytes were isolated from buffy coat and either grown to monocytes in MEM medium (1% peniciline/streptomycine, 1% L-Glutamine, 5% human serum) or differentiated to dendritic cells in medium supplemented with 50 ng/mL GM-CSF and 34 ng/mL IL-4. Cells were grown for 1 day at the air liquid interface with 1.2 mL RPMI 1640 complete medium in the lower well which was changed directly before aerosol exposure.

### Aerosol exposure experiments

For the here reported experiments, different ZnO aerosols were produced, by adjusting the runtime of the flame-spray synthesis reactor (22, 45, 90 sec). After particle production, the cell cultures were exposed to the glove-box atmosphere for 30 min, followed by a 4 or 24 h post-incubation period prior to sampling. For control experiments, the reactor was operated for 90 sec combusting only solvent w/o precursor (flame off-gases only), and cultures were exposed to the box atmosphere for 30 min followed by 4 or 24 h post-incubation.

### Suspension exposure experiments

A 1 mg/mL aqueous stock suspension was prepared by dispersing a flame-made ZnO powder in ultrapure water by the use of a UP400S probe sonicator (400 watt, Cycle 0.5, Power 100%, 5 min, volume ~ 25 mL, Hielscher Ultrasonics GmbH, Teltow, Germany). The suspensions were transported, shortly vortexed and further diluted in RPMI 1640 (w/o supplements) to final concentrations of 5, 15, 30, 60, and 80 ppm (= μg/mL). Cell cultures were exposed to 1 mL particle suspension, added to the upper well. The medium in the lower well was changed and reduced to 2 mL complete RPMI 1640.

### Deposition measurement in submerged A549 cell cultures

To determine particle deposition onto submerged cell cultures, depletion of the total zinc content within the cell culture supernatant was determined by AAS over time. Experiments were carried out with monocultures of the A549 alveolar (type II- like) epithelial cell line, obtained from the American Tissue Type Culture Collection (LGC Promochem, Molsheim, France). Cells were seeded at 1.1 × 10^6^ cells/well (area 3.8 cm^2^, volume 2 mL) in 12-well BD Falcon™ tissue culture plates (Becton Dickinson, Allschwil, Switzerland) and grown to confluence for 3 days. Different concentrations of ZnO suspensions were prepared as described previously, and cell cultures were exposed to 1 mL for various timespans up to 270 min. At each investigated timepoint, the supernatant was then carefully removed and diluted in 0.5 M acetic acid. The Zinc content was subsequently quantified by AAS as previously described.

To investigate the solubility of the used flame-made ZnO, a series of control experiments was performed [13,30]. ZnO suspensions were prepared in RPMI 1640 cell culture medium. After incubation under cell culture conditions (incubator) or on a shaker (1000/min) for various timespans, the particles were pelleted by centrifugation (maximal speed, 30 min, room temperature, VWR himac CT15E centrifuge; VWR International AG, Dietikon, Switzerland). The zinc content in the supernatant was determined by AAS and represents the dissolved (ionic) fraction of total zinc. The particle removing – efficiency of this process was controlled by UV-Visible Spectrophotometry of the supernatant after centrifugation (Shimadzu UV 1650PC, Shimadzu Schweiz GmbH, Reinach, Switzerland), using macro polymethylmethacrylate (PMMA) cuvettes (VWR International AG, Dietikon, Switzerland).

### Lactate dehydrogenase Release

As a general measure for cytotoxicity, the release of lactate dehydrogenase (LDH) from perforated cell cultures was assessed. The medium from the lower well (aerosol scenario) or upper and lower well (submerged cultures) was collected and stored at 4°C until analysis could occur. The commercially available LDH cytotoxicity detection kitPLUS (Roche Applied Science, Mannheim, Germany) was used according to the supplier’s manual. LDH was quantified photometrically by measuring at 490 nm, with 630 nm as reference wavelength. Each sample was assessed in triplicate and measured over time (kinetic). The values were expressed as absorbance at 5.5 min development time. A cell lysate served as positive control (Triton X-100 detergents, 30 min, 37°C).

### Chemokine/cytokine quantification

Inflammatory mediators (IL-8, TNFα, IL-1β, IL-6, MIP-1α) were quantified by the use of a Bio – Plex™ Cytokine Assay (6 – Plex Group I, Human) according to the supplier’s manual. Plates were analysed with a Bio – Plex 100 System (Bio-Rad Laboratories AG, Reinach, Switzerland).

### Total reduced gluthatione (GSH)

For sample preparation, an equal amount of an aqueous 50 mM 2-(*N*-morpholino)ethanesulfonic acid/1 mM Ethylenediaminetetraacetic acid solution (pH 6 – 7) was added to the medium contained within each well. Cell samples were then lysed using a cellscraper. The cell lysate was subsequently centrifuged (10,000 g, 15 min, 4°C) and an equivalent volume of 1.25 M metaphosphoric acid (MPA) was added. After incubation for 5 min at room temperature to allow deproteination to occur, the samples were centrifuged a second time (2000 g, 5 min, 4°C) and stored at – 80°C. The total GSH content was estimated by the glutathione assay kit (Cayman Chemical Company, Ann Harbor, U.S.A.), according to the suppliers manual. A standard in cell culture medium was used and 100 mM *tert*-Butylhydroperoxid (TBHP), known to induce GSH oxidation, served as positive control. The results were normalized to the total protein content of the particular samples, measured by a Pierce BCA Protein Assay Kit (Thermo Fisher Scientific, Lausanne, Switzerland).

### Real – time reverse – transcriptase polymer chain reaction (RT – PCR)

Following the post incubation period, insert membranes were cut out and transferred immediately into RNA protect buffer (Qiagen AG, Hombrechtikon, Switzerland) and stored at 4°C until analysis could occur. Cells were detached by vortexing and RNA was isolated by RNeasy plus kit (Qiagen AG) according to the manufacturer’s guidelines. RNA concentration was determined by a Nano Prop 2000 (Thermo Scientific AG, Wohlen, Switzerland) and samples were further diluted in water. Reverse transcription (incubation 1 h @ 37°C) was carried out in 10 μL volume with 0.25 μg/reaction template RNA, using a master – mix consisting of 0.25 mM of each dNTP (Qiagen AG), 0.5 μM Oligo dT primers (Qiagen AG), 10 units RNase inhibitor (RNasin Plus RNase Inhibitor, Promega AG, Dübendorf, Switzerland), 2 units Omniscript Reverse Transcriptase (Qiagen AG) and 1 × buffer RT (Qiagen AG). Real – time PCR was performed in a reaction volume of 10 μL, with a total of 2 μL of tenfold diluted cDNA, using a fast SYBR Green master mix with a 50 nM primer mix in a 7500 fast real-time PCR system (Applied Biosystems International Inc., Rotkreuz, Switzerland). Settings: Denature 20 sec @ 95°C, PCR Cycles (40): 3 sec @ 95°C, 30 sec @ 60°C. Relative expression levels were calculated using the ΔΔCt method with glyceraldehyde-3-phosphate reductase (GAPDH, GenBank NC_000012) as an internal reference gene. The expression levels of heme-oxygenase 1 (HMOX1, HO-1, GenBank CP002685) and superoxide dismutase 1 (SOD1, GenBank NM_000454) were determined. Primer (Microsynth AG, Balgach, Switzerland) sequences were the following: GAPDH: forward 5^′^- AAC AGC CTC AAG ATC ATC AGC-3^′^, reverse 5^′^- GGA TGA TGT TCT GGA GAG CC-3^′^; HMOX1: forward 5^′^- TTC TCC GAT GGG TCC TTA CAC T-3^′^, reverse 5^′^- GGC ATA AAG CCC TAC AGC AAC T-3^′^; SOD1: forward 5^′^- GTG CAG GTC CTC ACT TTA AT-3^′^, reverse 5^′^- CTT TGT CAG CAG TCA CAT TG-3^′^; IL-8: forward 5^′^- CTG GCC GTG GCT CTC TTG-3^′^, reverse 5^′^- CCT TGG CAA AAC TGC ACC TT-3′.

### Statistical analysis

Data were presented as mean ± standard deviation (SD). Unless otherwise stated, all biological analysis were performed a total of three times (n = 3). As normal distribution cannot be adequately tested based on such a small sample size, a non – parametric testing strategy was applied. Statistical analysis was performed with IBM SPSS Statistics 19 (Dynelytics AG, Zürich, Switzerland).

The influence of zinc dose on the investigated toxicological endpoints was assessed by a Friedman test for suspension - or a Kruska – Wallis test for aerosol - exposed samples (including gas control and all doses). Additionally, the Jonckheere - Terpstra group’s trend test was used to test for ordered alternative hypotheses (dose dependence) and was supplemented by the Kendall’s Tau test to correlate dose and response. Comparisons between 4 and 24 h (post –) incubation period was done by a Friedman test and pairwise differences among groups were analyzed by the Mann – Whitney *U* test. Values were considered as significant at *p<0.05*.

## Abbreviations

NP: Nanoparticle; ZnO: Zinc oxide; h: Hour; HO – 1: Heme oxygenase 1; SOD1: Superoxide dismutase 1; LDH: Lactate dehydrogenase; GSH: Glutathione; TNFα: Tumor necrosis factor – alpha; ALI: Air liquid interface; IL: Interleukin; MIP: Macrophage Inflammatory Protein; TNFα: Tumor Necrosis Factor alpha; ALI: Air Liquid Interface; AAS: Atomic absorption spectroscopy; FMPS: Fast mobility particle sizer; miniDISC: Portable diffusion size classifier; CPC: Condensation particle counter; SD: Standard deviation; Sec: Second; OD: Optical Density; OSHA: Occupational Safety and Health Administration; ROS: Reactive oxygen species; CO_2_: Carbon dioxide; CO: Carbon monoxide; NO_x_: Nitrogen oxides; RT – PCR: Real – time reverse – transcriptase polymer chain reaction.

## Competing interests

The authors declare no competing interests.

## Authors’ contributions

BR, WS and PG conceived the study and designed with RG, MJDC and DR the experiments. DR carried out the suspension exposure experiments and performed together with RG the aerosol exposure. DR analyzed the samples and wrote the manuscript. CS made the atomic absorption spectroscopy measurements. BR, MJDC and WS have intellectually accompanied the design of the study, the experimental work, made substantial contributions to the analysis and interpretation of the data, and have been involved in revising the manuscript critically for important intellectual content. All authors read and approved the final draft.

## Supplementary Material

Additional file 1 **Figure S1.** Heat maps representing the glove box aerosol size distribution in scenarios including 22 (identical to Figure 1B), 45 and 90 sec reactor operation. Chart S1. Matrix of Bio - Plex data. Beside LPS, also a TNFα control was performed. **Figure S2**. Decrease of zinc oxide concentration in the cellular supernatant over time. The first measurement was performed after 10 min incubation. **Figure S3**. Dynamic Light Scattering analysis of a 15 ppm ZnO suspension in RPMI 1640 cell culture medium (w/o supplements). **Figure S4**. Solubility of ZnO particles, dispersed in RPMI 1640, in dependence of time. Chart S2. Matrix of Bio - Plex data. Click here for file
