# Supplementary material for: Effects of flame made zinc oxide particles in human lung cells - a comparison of aerosol and suspension exposures
Source: Part Fibre Toxicol. 2012 Aug 17;9:33. doi: 10.1186/1743-8977-9-33 (PMC3585858; doi:10.1186/1743-8977-9-33)
Supplement: Additional file 1 — Figure S1. Heat maps representing the glove box aerosol size distribution in scenarios including 22 (identical to Figure 1B), 45 and 90 sec reactor operation. Chart S1. Matrix of Bio - Plex data. Beside LPS, also a TNFα control was performed. Figure S2. Decrease of zinc oxide concentration in the cellular supernatant over time. The first measurement was performed after 10 min incubation. Figure S3. Dynamic Light Scattering analysis of a 15 ppm ZnO suspension in RPMI 1640 cell culture medium (w/o supplements). Figure S4. Solubility of ZnO particles, dispersed in RPMI 1640, in dependence of time. Chart S2. Matrix of Bio - Plex data. [file 1743-8977-9-33-S1.pdf]

Supplementary Data

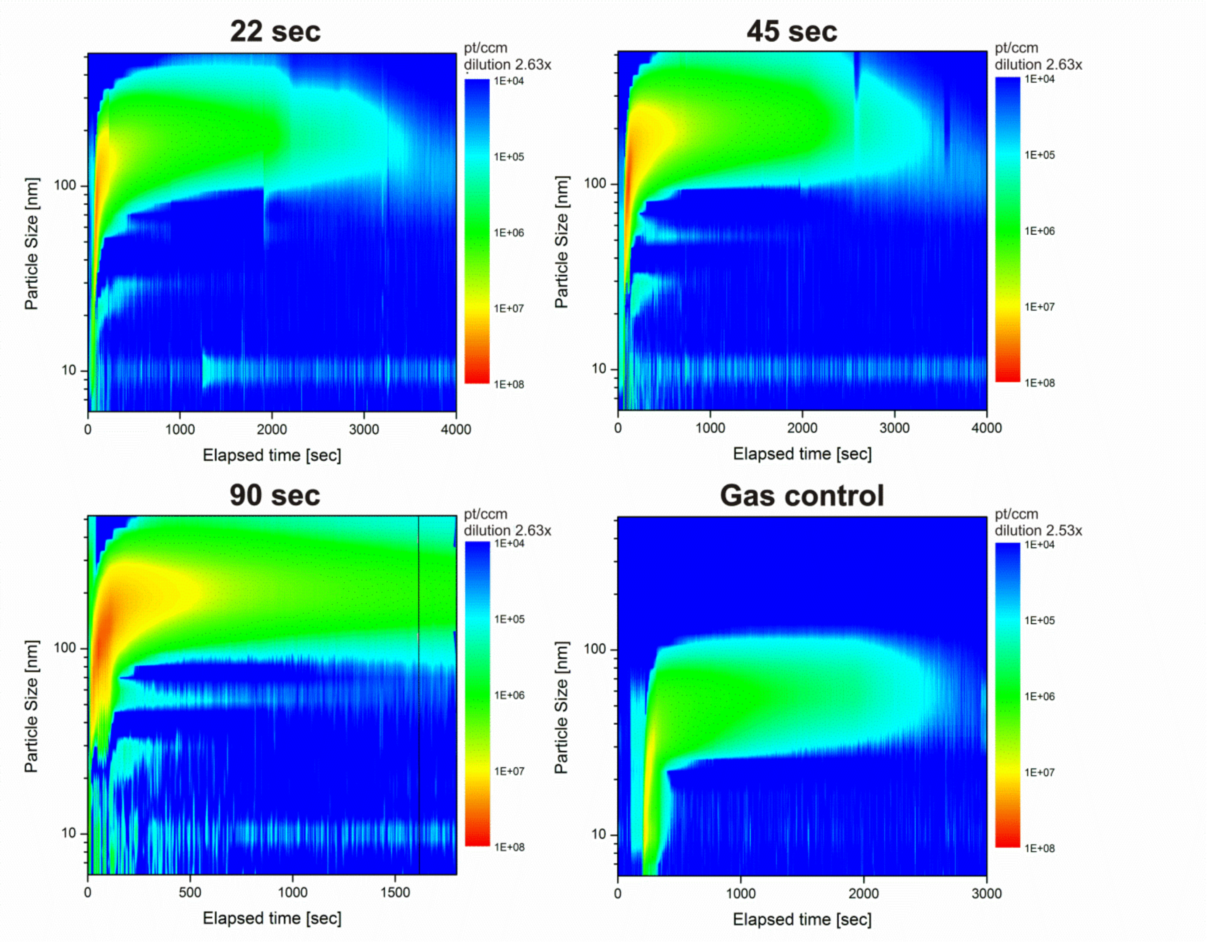

Fig. S1. Heat maps representing the glove box aerosol size distribution in scenarios including 22 (identical to Fig. 1B), 45 and 90 sec reactor operation. Additionally, a “gas control” was performed with 90 sec reactor runtime without precursor feed. A particle signal in the comparably small size range of ≤ 100 nm was detectable for the gas control as well. This may be explained by the formation of droplets due to condensation of H_2O, originated from the combustion process, or by incomplete burning / oxidation of the solvent. The time resolution of all FMPS measurements was 1 sec. The blue color indicates values ≤1e4 pt / ccm.

Bio – Plex Cytokine Assay: Aerosol Experiments

4 h Post-Incubation	TNFα		MIP-1α		IL-10		IL-1β	
	Avg	SD	Avg	SD	Avg	SD	Avg	SD
**Incubator Control**	1058.1	260.8	789.1	491.2	5.3	2.1	30.4	5.3
**Gas-Control (90sec)**	4048.6	629.8	725.1	400.7	4.8	1.5	43.1	7.1
**22 sec**	2381.6	1355.6	397.7	181.7	4.0	2.5	52.0	15.5
**45 sec**	3241.1	1122.7	583.7	293.8	5.6	1.8	53.2	3.2
**90 sec**	2765.7	621.3	321.6	123.6	3.5	0.4	45.5	7.4
**LPS**	9438.3	4906.7	3232.1	1877.2	22.0	5.7	308.4	74.0
**TNFα**	129851.3	19637.6	2256.7	1545.4	18.7	3.8	225.0	26.4

24 h Post-Incubation	TNFα		MIP-1α		IL-10		IL-1β	
	Avg	SD	Avg	SD	Avg	SD	Avg	SD
**Incubator Control**	1334.9	459.9	1157.7	510.5	5.8	3.5	37.0	3.0
**Gas-Control (90sec)**	3967.6	1639.8	1294.6	191.5	6.5	3.3	59.6	18.0
**22 sec**	3851.1	1457.8	587.9	271.3	4.5	2.6	51.9	0.6
**45 sec**	4722.1	2715.9	914.2	226.4	5.2	2.4	60.2	13.8
**90 sec**	3126.8	1209.3	734.3	83.6	4.7	1.8	55.4	12.9
**LPS**	7841.1	1580.8	3793.5	1012.2	20.2	6.0	366.6	42.5
**TNFα**	132130.7	33730.4	2588.5	1151.3	15.5	2.6	295.4	58.8

Values are expressed as pg / mL

Chart S1. Matrix of Bio - Plex data. Beside LPS, also a TNFα control was performed.

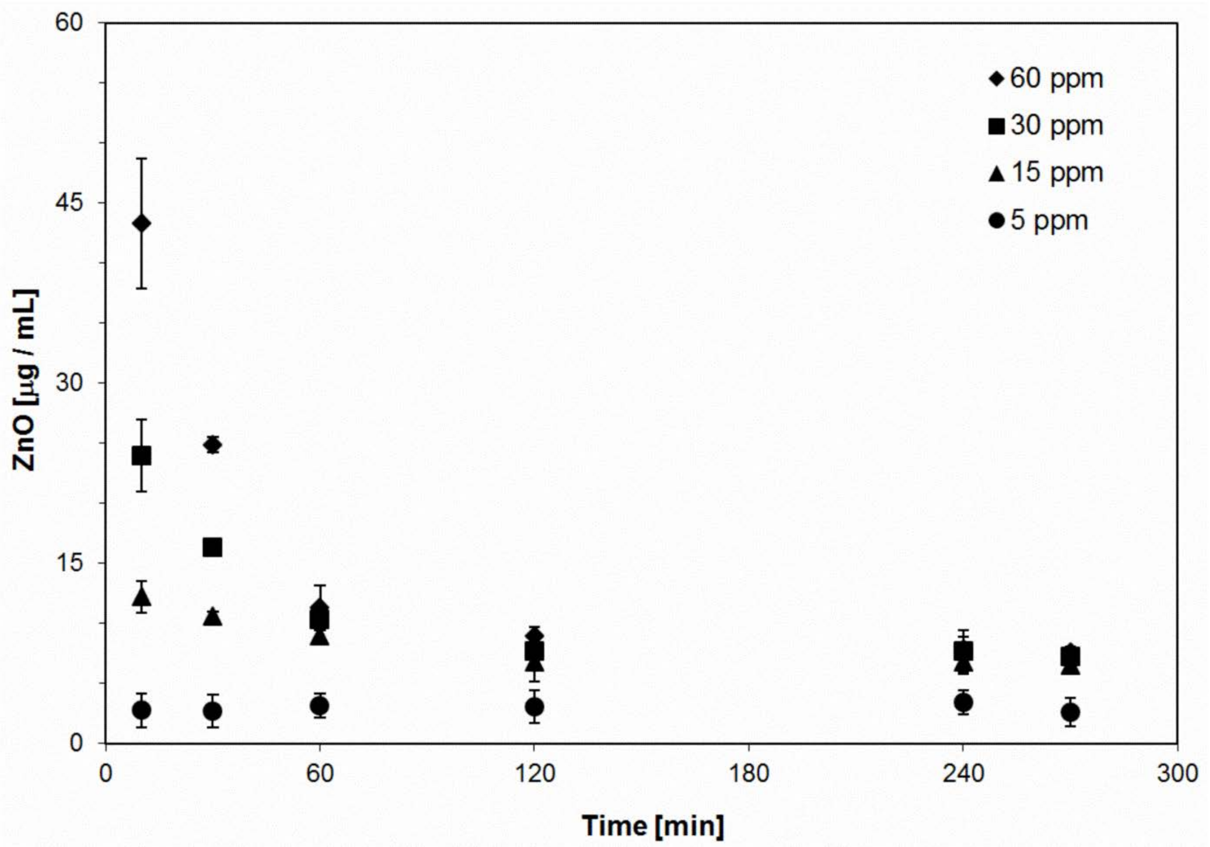

Fig. S2. Decrease of zinc oxide concentration in the cellular supernatant over time. The first measurement was performed after 10 min incubation. The values decrease over the first 120 min and reach a plateau. After 240 min, 3.4 (SD 1.0, n = 4), 6.7 (SD 1.0, n = 4), 7.7 (SD 1.8, n = 4) and 7.8 (SD 1.1, n = 4) µg /mL were measured in 5 – 60 ppm suspensions. This corresponds to 68.6, 44.9, 25.6 and 13.0 % of the initial concentration.

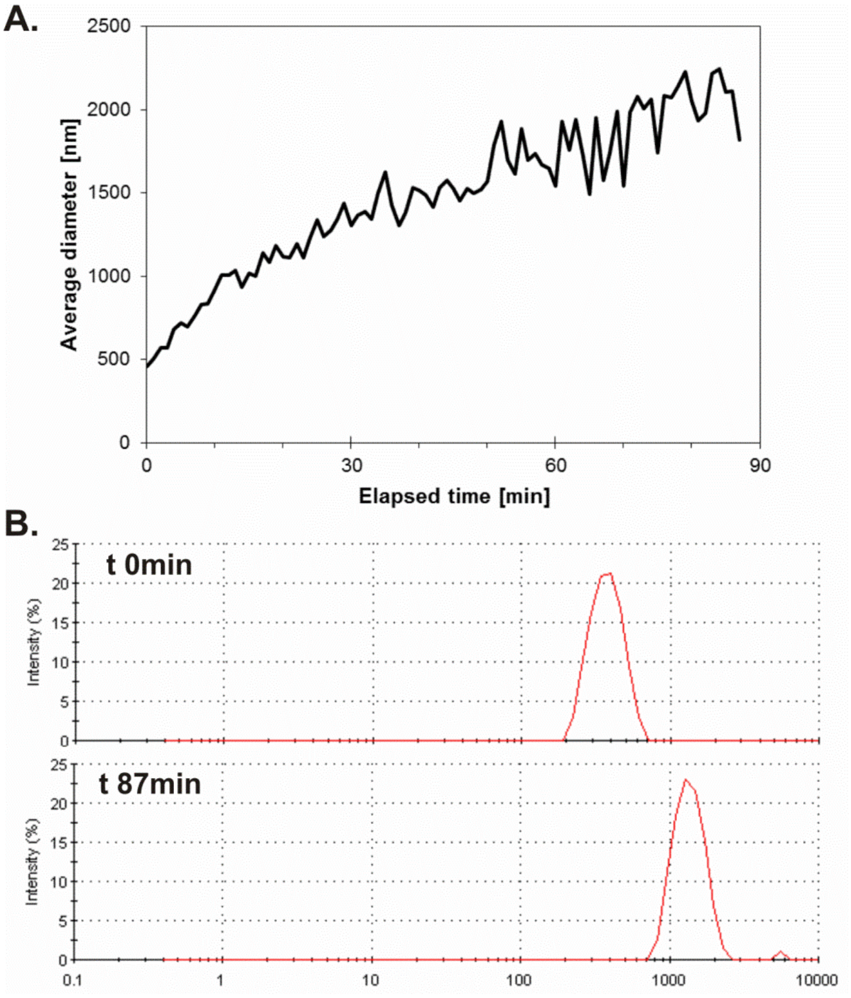

Fig. S3. Dynamic Light Scattering analysis of a 15 ppm ZnO suspension in RPMI 1640 cell culture medium (w / o supplements). **A.** Evolution of the Z – average particle diameter over time. **B.** Corresponding size distribution by intensity. Hydrodynamic diameter is given in nm. Results were derived from one single experiment.

Particle size distribution was measured by Dynamic Light Scattering with the following instrument settings: Water as dispersant, 25 °C, 173° scattering angle. Zinc oxide suspensions were prepared as descried in materials and methods.

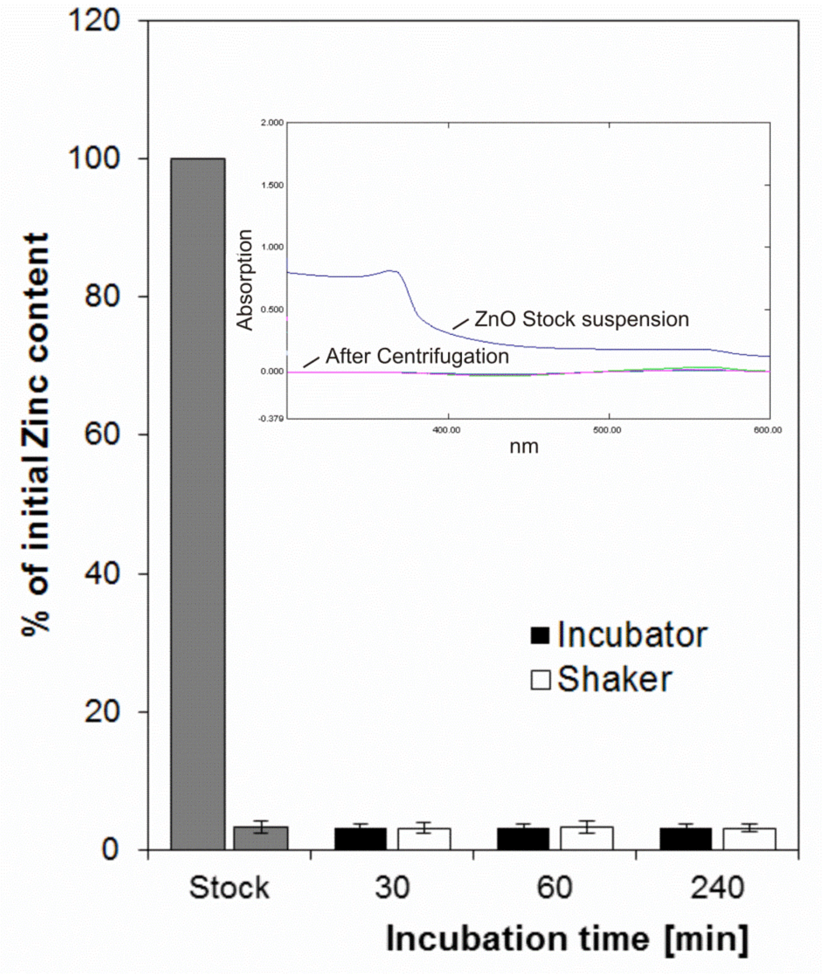

Fig. S4. Solubility of ZnO particles, dispersed in RPMI 1640, in dependence of time. After incubation under cell culture conditions (incubator) or on a shaker for various timespans, the particles were pelleted by centrifugation, and the zinc content in the supernatant was determined by AAS. This dissolved (ionic) fraction of total Zn was expressed relative to the total zinc content of the stock suspension. A solubility in the range of 4 % was observed over all time points, what is comparable with values in the literature. No difference between a static and a vigorously stirred environment can be found and dissolution is independent of time.

To validate the used particle – separation protocol, control experiments were performed. The inset shows a representative uv-vis spectrum taken either form an untreated ZnO stock suspension, or from the supernatant after spinning. The characteristic zinc oxide peak disappears with centrifugation, indicating a supernatant free of particles with this procedure.

Bio – Plex Cytokine Assay: Suspension experiments

4 h Incubation

Apical	TNFα		MIP-1α		IL-10		IL-8		IL-6		IL-1β	
	Avg	SD	Avg	SD	Avg	SD	Avg	SD	Avg	SD	Avg	SD
Control	160.1	10.4	104.9	97.8	0.5	0.1	922.8	512.6	1036.9	158.5	5.5	3.2
5 ppm	319.5	80.7	93.8	94.5	0.4	0.2	927.0	465.2	1044.6	125.9	4.7	3.4
15 ppm	365.8	71.2	65.1	58.3	0.4	0.1	845.1	202.4	1080.7	141.5	3.8	2.5
30 ppm	360.8	45.7	51.6	40.3	0.2	0.2	691.8	205.6	936.9	73.9	3.0	1.7
60 ppm	447.7	60.7	54.0	50.3	0.3	0.1	625.3	139.9	947.7	239.7	3.1	1.7
80 ppm	396.1	55.2	53.3	37.0	0.3	0.1	504.0	109.6	990.1	221.7	3.1	1.4
LPS	270.8	105.1	54.3	26.6	0.4	0.0	519.8	71.6	1014.4	435.7	4.7	1.6

Basal	TNFα		MIP-1α		IL-10		IL-8		IL-6		IL-1β	
	Avg	SD	Avg	SD	Avg	SD	Avg	SD	Avg	SD	Avg	SD
Control	190.2	38.1	114.1	64.2	0.9	0.3	1880.3	600.9	1195.5	373.8	7.1	1.8
5 ppm	225.7	50.8	110.3	45.7	0.9	0.5	1578.0	537.4	1063.1	200.0	8.1	1.3
15 ppm	204.4	45.5	101.3	35.2	1.1	0.7	1767.8	715.3	1024.2	196.0	7.6	1.5
30 ppm	204.4	42.0	120.8	57.8	1.5	1.1	*1423.9	*405.7	1117.1	294.4	8.2	1.9
60 ppm	222.6	41.1	112.2	47.9	0.8	0.3	1703.9	509.7	1150.2	280.7	7.7	1.5
80 ppm	196.5	42.7	101.5	49.6	0.6	0.2	1639.9	385.5	1010.8	209.6	6.6	2.1
LPS	228.4	122.6	69.2	32.7	*1.1	*1.3	828.4	387.1	727.2	452.3	4.1	1.1

24 h Incubation

Apical	TNFα		MIP-1α		IL-10		IL-8		IL-6		IL-1β	
	Avg	SD	Avg	SD	Avg	SD	Avg	SD	Avg	SD	Avg	SD
Control	363.9	84.5	388.1	281.6	1.5	0.3	4865.8	2113.4	3937.0	1996.8	12.5	2.4
5 ppm	632.2	261.5	367.4	133.9	1.1	0.3	*6693.6	*925.6	*4012.5	*69.5	12.6	2.1
15 ppm	773.1	136.2	147.9	12.8	0.7	0.2	5878.1	2315.0	5141.4	2006.7	5.7	0.4
30 ppm	830.8	201.3	126.1	24.9	0.7	0.1	3475.9	378.5	4135.0	1363.4	5.1	0.6
60 ppm	1010.1	185.3	128.5	61.1	0.5	0.3	2976.1	1003.4	4133.5	1618.7	4.9	1.0
80 ppm	993.4	251.6	121.2	61.0	0.4	0.2	2223.8	619.5	3182.2	1291.8	5.0	1.3
LPS	1833.5	1057.4	*543.0	*138.7	*1.7	*0.0	*7464.6	*1084.6	7741.5	86.8	90.5	67.3

Basal	TNFα		MIP-1α		IL-10		IL-8		IL-6		IL-1β	
	Avg	SD	Avg	SD	Avg	SD	Avg	SD	Avg	SD	Avg	SD
Control	271.9	63.0	*503.34	*177.9	2.1	0.3	*8090.0	*2212.6	6339.0	5236.1	12.8	1.5
5 ppm	350.7	125.1	434.6	72.6	2.3	1.3	*7010.7	*2525.0	3581.4	820.3	13.1	1.9
15 ppm	375.6	75.8	490.2	158.7	1.9	0.7	*9038.9	*2582.1	3250.1	328.1	13.2	1.3
30 ppm	403.1	54.8	404.3	138.4	2.0	1.3	9252.4	2096.1	3298.0	502.8	12.9	0.8
60 ppm	439.2	100.6	462.0	280.1	2.0	0.6	*4722.4	*1059.1	3466.6	1308.3	13.1	1.7
80 ppm	468.5	136.0	430.0	215.3	2.2	0.8	*7408.1	*2242.3	3090.8	1367.3	13.5	1.0
LPS	1421.7	476.8	613.1	12.5	*1.7	*0.4	10873.3	264.6	*13473.4	*5510.8	46.0	20.6

Values are expressed as pg / mL

* indicates a N = 2

Chart S2. Matrix of Bio - Plex data.
